# Supplementary material for: The 3 × 2 Achievement Goals in the Education, Sport, and Occupation Literatures: A Systematic Review with Meta-Analysis
Source: Eur J Investig Health Psychol Educ. 2023 Jun 21;13(7):1130–57. doi: 10.3390/ejihpe13070085 (PMC10416154; doi:10.3390/ejihpe13070085)
Supplement: Supplementary file 1 [file ejihpe-13-00085-s001.zip › ejihpe-2412229-supplementary-update.pdf]

Supplemental File. Search results.

Database	Search Term	Time	Sort by	Yes	Duplication	Criteria1	Criteria2	Criteria3	Initial Screening	Article citation
Google Scholar	"3x2 achievement goal model"	any	relevance							
				x					yes	Lower, L. M., & Turner, B. A. (2016). Examination of the 3x2 achievement goal model in collegiate recreation: Comparison across sport programs. Journal of Amateur Sport, 2(2), 75-102.
				x					yes	Thomas, C. L. (2021). Predicting test anxiety using the 3x2 achievement goal model. International Journal of School & Educational Psychology, 1-11.
				x					yes	Lower, L. M., Newman, T. J., Pollard, W. S., & Lower, L. (2016). Examination of the 3x2 achievement goal model in recreational sport: Associations with perceived benefits of sport participation. International Journal of Sport Management, Recreation & Tourism, 26, 44-53.
				x					yes	YERDELEN, S., & PADIR, M. A. (2017). Adaptation of 3x2 achievement goal questionnaire for teachers into Turkish: Validity and reliability study. Bartın University Journal of Faculty of Education, 6(3), 1027-1039.
				x					yes	Lüftenegger, M., Klug, J., Harrer, K., Langer, M., Spiel, C., & Schober, B. (2016). Students' achievement goals, learning-related emotions and academic achievement. Frontiers in psychology, 7, 603.
				x					yes	Nikitskaya, M. G., & Uglanova, I. L. (2021). The Russian Version of the Educational Achievement Goal Questionnaire: Development, Validation and Research of Functionality. Psychological Science and Education, 26(5), 67-84.
						x			no, no 3x2	Brockbank, R. D., Smith, D. T., & Oliver, E. J. (2020). Dispositional goals and academic achievement: refining the 2x2 achievement goal model. Sport and exercise psychology review., 16(1).
				x					yes	Cecchini, J. A., Méndez-Giménez, A., & García-Romero, C. (2021). Intra-individual changes in 3x2 achievement goals, friendship goals, motivational regulations and consequences in physical education. Revista Latinoamericana de Psicología, 53, 180-189.
						x			no, no 3x2	Sarıcı, E., & Kondakçı, E. (2016). Investigating the role of students goal orientations on their understanding of chemical equilibrium concepts.
				x					yes	Luo, S., Gan, J. S., Loh, A. P., Ee, E. W. H., & Soh, E. K. (2019, December). Evaluating Student Academic Motivations in One-year CubeSat Project using 3x 2 Achievement Goal Framework. In 2019 IEEE International

										Conference on Engineering, Technology and Education (TALE) (pp. 1-7). IEEE.
							x		no, citation	Uglanova, I. L., & Nikitskaya, M. G. Russian adaptation of the test based on 3x2 Achievement Goal Model (Elliot, Murayama, Pekrun, 2011). 2021. Mendeley Data, 1.
							x		no, citation	Elliot, A. J., & Kou, M. Pekrun Reinhard,(2011).“A 3x2 achievement goal model”. Journal of Educational Psychology, 1033, 632-648.
				x					yes	Altmeyer, M., Lessel, P., Waqar, A. U. R., & Krüger, A. (2021). Design guidelines to increase the persuasiveness of achievement goals for physical activity. arXiv preprint arXiv:2107.12599.
				x					yes	García-Romero, C., Méndez-Giménez, A., & Cecchini-Estrada, J. A. 3X2 ACHIEVEMENT GOALS AND PSYCHOLOGICAL MEDIATORS IN PHYSICAL EDUCATION STUDENTS METAS DE LOGRO 3X2 Y MEDIADORES PSICOLÓGICOS EN ESTUDIANTES DE EDUCACIÓN FÍSICA.
				x					yes	Üztemur, S. (2020). Achievement goals and learning approaches in the context of social studies teaching: Comparative analysis of 3x2 and 2x2 models. Participatory Educational Research, 7(2), 1-18.
							x		no, not journal	Siu-Man, N. D., & Leung, M. T. (2014, May). A path analytic model of Chinese-style achievement motivation, 3× 2 achievement goals and self-regulated learning of Hong Kong undergraduates. In 2014 Asian congress of applied psychology: Conference proceedings (pp. 292-319).
				x					yes	Méndez Giménez, A., García Romero, C., & Cecchini Estrada, J. A. (2018). 3x2 Achievement goals, friendship and affectivity in physical education: age-gender differences. Revista Internacional de Medicina y Ciencias de la Actividad Física y del Deporte, 18.
					x				dupe	Yerdelen, S., & Padir, M. A. (2017). Öğretmenler için 3x2 başarı yönelimi ölçeği'nin Türkçeye uyarlanması: Geçerlilik-güvenirlik çalışması. Bartin Üniversitesi Egitim Fakültesi Dergisi, 6(3), 1027-1039.
				x					yes	Méndez-Giménez, A., Cecchini-Estrada, J. A., Fernández-Río, J., Saborit, J. A. P., & Méndez-Alonso, D. (2017). 3x2 classroom goal structures, motivational regulations, self-concept, and affectivity in secondary school. The Spanish Journal of Psychology, 20.
				x					yes	So, C. Y., & Leung, M. T. (2016). Structural equation modeling of Chinese parenting predicting Hong Kong secondary school students' learning strategies with achievement emotions and achievement goals as mediators. In Applied Psychology: Proceedings of the 2015 Asian Congress of Applied Psychology (ACAP 2015) (pp. 220-238).
				x					yes	Øvretveit, K., Sæther, S. A., & Mehus, I. (2019). Mastery goals are associated with training effort in Brazilian jiu-jitsu.

							x		no, not journal	Thompson, K., Craig, B., Leventhal, B. C., & Horst, S. J. Let's Talk About Attitudes: What Predicts First-Year Oral Communication Competence?. SAT, 1(3.26), 208-87.
				x					yes	Wu, C. C. (2022). Investigating the Discriminant Utility of Task-Based and Self-Based Goals in 3× 2 Achievement Goal Model for Kindergarteners. Children, 9(11), 1765.
					x				dupe	Méndez-Giménez, A., García-Romero, C., & Cecchini-Estrada, J. A. ACHIEVEMENT GOALS 3x2, FRIENDSHIP AND AFFECTIVITY IN PHYSICAL EDUCATION: AGE-GENDER DIFFERENCES METAS DE LOGRO 3x2, AMISTAD Y AFECTO EN EDUCACIÓN FÍSICA: DIFERENCIAS EDAD-SEXO.
							x		no, not journal	Dudkina, A., & Klekmane, K. (2016, May). STUDENTS ACHIEVEMENT GOALS GENDER DIFFERENCES. In SOCIETY. INTEGRATION. EDUCATION. Proceedings of the International Scientific Conference (Vol. 1, pp. 320-330).
				x					yes	Didin, M., & Kasapoglu, K. (2021). Seventh Graders' Learning Strategies and Achievement Goal Orientations as Predictors of Their Achievement in Social Studies. International Journal of Progressive Education, 17(3), 361-380.
							x		no, not journal	Janse, R. (2016). Personality and achievement goals Relating the HEXACO Personality Factors to the 3 x 2 Achievement Goal Model (Master's thesis, University of Twente).
					x				dupe	Méndez-Giménez, A., García-Romero, C., & Cecchini-Estrada, J. A. EDUCACIÓN FÍSICA: DIFERENCIAS EDAD-SEXO.
							x		no, not journal	Ansems, I. E. L. Mastery Motivation in Measurement Feedback.
					x				dupe	García-Romero, C., Méndez-Giménez, A., & Cecchini-Estrada, J. A. METAS DE LOGRO 3X2 Y MEDIADORES PSICOLÓGICOS EN ESTUDIANTES DE EDUCACIÓN FÍSICA 3X2 ACHIEVEMENT GOALS AND PSYCHOLOGICAL MEDIATORS IN PHYSICAL EDUCATION STUDENTS.
				x					yes	Hidayat, R., Zamri, S. N. A. S., & Zulnaidi, H. (2018). Exploratory and confirmatory factor analysis of achievement goals for Indonesian students in mathematics education programmes. EURASIA Journal of Mathematics, Science and Technology Education, 14(12), em1648.
						x			no, no 3x2	Menon, R., & Mokhtar, A. H. (2017). Cross-Cultural Validation of the Achievement Goal Questionnaire for Sports: A Malaysian Adaptation. Available at SSRN 2924348.
						x			no, no 3x2	O'Keefe, P. A., Ben-Eliyahu, A., & Linnenbrink-Garcia, L. (2013). Shaping achievement goal orientations in a mastery-structured environment and concomitant changes in related contingencies of self-worth. Motivation and emotion, 37(1), 50-64.

						x			no, no 3x2	Korn, R. M., & Elliot, A. J. (2016). The 2× 2 standpoints model of achievement goals. Frontiers in psychology, 7, 742.
							x		no, not journal	Hidayat, R., Zamri, S. N. A. S., Zulnaidi, H., & Yuanita, P. (2020). Achievement Goal for Indonesian Students of Mathematics Education Program: Issues of Gender and Academic Yeal Level. Revolution, 4, 492-498.
				x					yes	Hunsu, N., Oje, A. V., Jackson, A., & Olaogun, O. P. (2021). Examining Approach and Avoidance Valences of the 3 X 2 Achievement Goal Types on an Engineering Student Sample: A Validity Approach. Frontiers in Psychology, 12, 628004.
							x		no, not journal	Wilhelmsson, M. (2013). A personalized achievement system for educational games: Targeting the achievement goals of the student.
							x		no, not journal	Awawdi, H. (2019). The Consequences of Promoting Mastery Goal Using Autonomy Supportive and Controlling Practices in the Classroom (Doctoral dissertation, University of Haifa (Israel)).
				x					yes	أنور عبد الغني, إ., & محمد سعيد, ن. (2018). النمذجة السببية لتوجهات أهداف الإنجاز (النموذج السداسي 3X2) والاندماج المعرفي والتحصيل الأكاديمي في ضوء متغيري النوع والتخصص. مجلة كلية التربية (أسيوط), 34(3), 1-83.
				x					yes	Gegenfurtner, A. (2019). Reconstructing goals for transfer of training in faculty development programs for higher education teachers: A qualitative documentary method approach. Heliyon, 5(11), e02928.
						x			no, 2x2	Miller, A. L. Achievement Goal Orientation as a Predictor of High-Impact Practice Participation for Postsecondary Students. Mid-Western Educational Researcher, 34(3), 223.
							x		no, dissertation	D'Astous, E. G. (2016). An examination of Elliot's hierarchical model of approach and avoidance achievement motivation in athletes returning to sport following serious injury. The University of Utah.
						x			no, 2x2	Oliver, E. M of interest in high-performance.
				x					yes	León-del-Barco, B., Mendo-Lázaro, S., Polo-del-Río, M. I., & Rasskin-Gutman, I. (2019). University Student’s Academic Goals When Working in Teams: Questionnaire on Academic Goals in Teamwork, 3× 2 Model. Frontiers in psychology, 10, 2434.
				x					yes	Beretta, A., Zanetti, M. A., & Renati, R. (2013). Metacognizione, obiettivi di apprendimento e successo scolastico in studenti ad alto potenziale della scuola superiore. Metacognizione, obiettivi di apprendimento e successo scolastico in studenti ad alto potenziale della scuola superiore, 353-370.
							x		no, thesis	Mees, A., & Jonckheere, K. WANNEER LEADER-MEMBER EXCHANGE DE JOBTEVREDENHEID VERSTERKT: DE MODERERENDE ROL VAN ACHIEVEMENT GOAL ORIENTATION.

					x				dupe	Cornillie, F., Lagatie, R., Vandewaetere, M., Clarebout, G., & Desmet, P. (2013). Tools that detectives use: in search of learner-related determinants for usage of optional feedback in a written murder mystery. CALICO Journal, 30, 22-45.
						x			no, no 3x2	Miller, A. L., & Speirs Neumeister, K. L. (2017). The influence of personality, parenting styles, and perfectionism on performance goal orientation in high ability students. Journal of Advanced Academics, 28(4), 313-344.
				x					yes	Abercrombie, S., Bang, H., & Vaughan, A. (2022). Motivational and disciplinary differences in academic risk taking in higher education. Educational Psychology, 1-18.
				x					yes	Hidayat, R., Zulnaidi, H., & Syed Zamri, S. N. A. (2018). Roles of metacognition and achievement goals in mathematical modeling competency: A structural equation modeling analysis. PloS one, 13(11), e0206211.
									no, review article	Martin, A. J. (2015). Growth approaches to academic development: Research into academic trajectories and growth assessment, goals, and mindsets. British Journal of Educational Psychology, 85(2), 133-137.
				x					yes	Cornillie, F., & Desmet, P. (2013). *Seeking out fun failure: How positive failure feedback could enhance the instructional effectiveness of CALL mini-games. Global perspectives on Computer-Assisted Language Learning. Proceedings of WorldCALL 2013, 64-68.*
							x		no, thesis	Tsai, H. J. (2015). Exploring college students' motivational beliefs in ability-grouped English classes in Taiwan (Doctoral dissertation, Durham University).
							x		no, thesis	Özuzun, Y. B. (2018). Differentiation of dual motivational system in deviance regulation theory by achievement goal orientations (Master's thesis, Middle East Technical University).
						x			no, no 3x2	Holden, S. M., Mueller, C. E., Harrell-Williams, L. M., Ford, J. M., & Jones, M. H. (2021). Comparison of motivational latent profiles using the PALS and AGQ-R. Contemporary Educational Psychology, 67, 101999.
				x					yes	Méndez-Giménez, A., Cecchini-Estrada, J. A., & Fernández-Río, J. (2014). Examinando el modelo de metas de logro 3x2 en el contexto de la Educación Física. Cuadernos de Psicología del Deporte, 14(3), 157-168.
				x					yes	Lower-Hoppe, L. M., Evans, J. O., & Brgoch, S. M. (2021). Examining the social cognitive determinants of collegiate recreational sport involvement and outcomes. Leisure/Loisir, 45(2), 207-236.
							x		no, not journal	Bernardo, A. B. (2018). Sociocultural dimensions of student motivation: Research approaches and insights from the Philippines. Asian Education Miracles, 139-154.

							x		no, not journal	Parton, L. B. N. (2016). THE RELATIONSHIP BETWEEN SERVANT LEADER COACH BEHAVIORS AND ACHIEVEMENT GOALS IN COLLEGIATE TENNIS PLAYERS: THE MEDIATINGEFFECT OF MOTIVATIONAL CLIMATE.
						x			no, no 3x2	Dekker, S., Krabbendam, L., Lee, N., Boschloo, A., De Groot, R., & Jolles, J. (2016). Dominant goal orientations predict differences in academic achievement during adolescence through metacognitive self-regulation. Journal of Educational and Developmental Psychology, 6(1), 47-58.
							x		no, not journal	Hollis, R. B. (2013). Mind wandering and online learning: A latent variable analysis. Kent State University.
							x		no, not journal	Lowe, A. N. (2020). Identity Safety and Its Importance for Academic Success. Handbook on promoting social justice in education, 1849-1881.
						x			no, no 3x2	Woldemichael, B. B., Semela, T., & Tulu, A. (2022). THE EFFECT MASTERY AND PERFORMANCE RELATED MATHEMATICS LEARNING MOTIVATION ON MATHEMATICS ACHIEVEMENT: THE CASE OF FIRST YEAR UNDERGRADUATE UNIVERSITY STUDENTS IN BONGA UNIVERSITY, ETHIOPIA. International Journal of Education, Technology and Science, 2(4), 429-454.
								x	no, not correct use of 3x2	Putwain, D., Symes, W., Nicholson, L., & Becker, S. Achievement goals, behavioural engagement, and mathematics achievement.
				x					yes	Hidayat, R., Zamri, S. N. A. S., & Zulnaidi, H. (2018). Does mastery of goal components mediate the relationship between metacognition and mathematical modelling competency?. Educational Sciences: Theory & Practice, 18(3).
					x				dupe	Méndez-Giménez, A., García-Romero, C., & Cecchini-Estrada, J. A. (2018). Metas de logro 3x2, amistad y afecto en educación física: Diferencias edad-sexo. Revista Internacional de Medicina y Ciencias de la Actividad Física y el Deporte.
				x					yes	García Romero, C., Méndez Giménez, A., & Cecchini Estrada, J. A. (2020). Papel predictivo de las metas de logro 3x2 sobre la necesidad de autonomía en Educación Física. Sportis-Scientific Technical Journal Of School Sport Physical Education And Psychomotricity, 6.
				x					yes	الملاحة, ح. (2020). الذكاء الناجح و توجهات أهداف الانجاز كمنبئات باستراتيجيات مواجهة الضغوط الأكاديميةلدى طلبة الكليات الطبية مرتفعي و منخفضي التحصيل. مجلة جامعة الفيوم للعلوم التربوية والنفسية, 14(10), 63-127.
				x					yes	Leenknecht, M., Hompus, P., & van der Schaaf, M. (2019). Feedback seeking behaviour in higher education: the association with students' goal orientation and deep learning approach. Assessment & Evaluation in Higher Education, 44(7), 1069-1078.

								x	no, review article	Durmić, A. (2020). Achievement Goals: Conceptual Models and Results of Researching the Outcomes. Croatian Journal of Education: Hrvatski časopis za odgoj i obrazovanje, 22(1), 115-141.
							x		no, conference proceeding	Biddle, E., Lameier, E., Reinerman-Jones, L., Matthews, G., & Boyce, M. (2018, May). Personality: A key to Motivating our Learners. In 6th Annual GIFT Users Symposium (pp. 9-11).
							x		no, not journal	Delrue, J. (2018). Towards a more refined and integrative view on athletes' motivation and coaches' motivating style (Doctoral dissertation, Ghent University).
								x	no, no quantitative data	Irvine, J. (2018). A Framework for Comparing Theories Related to Motivation in Education. Research in Higher Education Journal, 35.
							x		no, thesis	Porras Rojas, P. S. (2021). Escala metas de logro 3x2 en estudiantes de secundaria de un colegio de Lima Metropolitana: evidencias de validez y confiabilida.
					x				dupe	Никитская, М. Г., & Угланова, И. Л. (2021). Русскоязычная версия опросника целей учебных достижений: разработка, валидизация и исследование функциональных возможностей. Психологическая наука и образование, 26(5).
					x				dupe	García-Romero, C., Méndez-Giménez, A., & Cecchini-Estrada, J. A. (2021). Metas de logro 3x2 y mediadores psicológicos en estudiantes de educación física. Revista Internacional de Medicina y Ciencias de la Actividad Física y del Deporte, 22(87), 455-469.
							x		thesis	Hannan, G. V. (2016). Girls' perceptions of challenging work and the factors that motivate them to engage with challenging work within the selective independent sector (Doctoral dissertation, UCL (University College London)).
						x			no, no 3x2	ALPARSLAN, A., ÖZKUL, A. S., & ERHAN, T. (2021). Düşünme İhtiyacı–Ustalık Yönelimi-Psikolojik İyi Oluş İlişkisi: Eğitimciler Üzerinde Bir İnceleme. Yönetim Bilimleri Dergisi, 19(42), 969-990.
								x	no, no quantitative data	Короткевич, Э. Р. (2019). Учебная мотивация современного студента в контексте теории достижения целей. Образовательные ресурсы и технологии, (3 (28)), 27-32.
							x		thesis	Marjanovic, M. (2014). The relationships between achievement goals, motivational climate and self-talk in physical education (Master's thesis).
							x		thesis	Kamarova, S. (2016). On the social nature of competence evaluations: Do task-involved individuals compare themselves to others? (Doctoral dissertation, Curtin University).

				x					yes	مصطفى, ف. م. م., & فتحي محمد محمود. (2021). توجهات أهداف الانجاز (2× 3) كمنبئات بالسلوكيات الأكاديمية المرتبطة بالوقت لدى طلاب جامعة القصيم. مجلة كلية التربية بالمنصورة, 116(2), 749-795.
							x		citation	Nikitskaya, M. G. (2019). Study on Achievement Goals and Directionality in the Context of Learning Motivation. Journal: Современная зарубежная психология Journal of Modern Foreign Psychology, (2), 26-35.
					x				dupe	Méndez-Giménez, A., García-Romero, C., & Cecchini-Estrada, J. A. METAS DE LOGRO 3x2, AMISTAD Y AFECTO EN EDUCACIÓN FÍSICA: DIFERENCIAS EDAD-SEXO ACHIEVEMENT GOALS 3x2, FRIENDSHIP AND AFFECTIVITY IN PHYSICAL EDUCATION: AGE-GENDER DIFFERENCES.
						x			no, no 3x2	Madjar, N., Ratelle, C. F., & Duchesne, S. (2021). A longitudinal analysis of the relationships between students' internalized symptoms and achievement goals. Motivation Science.
							x		citation	Quick, J. M. 1. Leveraging Proven Research in Educational Game Design.
				x					yes	Romero, C. G. (2022). Relación entre las metas de logro 3x2, las necesidades psicológicas básicas y la inteligencia emocional en estudiantes de Educación Física. In Congreso EDUCA 2022: Ebook de Actas. 5ª Congreso Mundial de Educación 24-26 de febrero, 2022 (pp. 423-425). Campus Educa-Sportis.
						x			no 3x2	Josefsson, T., Ivarsson, A., Gustafsson, H., Stenling, A., Lindwall, M., Tornberg, R., & Böröy, J. (2019). Effects of mindfulness-acceptance-commitment (MAC) on sport-specific dispositional mindfulness, emotion regulation, and self-rated athletic performance in a multiple-sport population: an RCT study. Mindfulness, 10(8), 1518-1529.
						x			no, no 3x2	Bipp, T., Kleingeld, A., & Schelp, L. (2021). Achievement goals and goal progress as drivers of work engagement. Psychological Reports, 124(5), 2180-2202.
							x		thesis	Berger, N. (2013). Exploring the motivational goals of Preliminary HSC students from divergent socioeconomic backgrounds. Unpublished master's thesis), The University of Newcastle, Callaghan, Australia. https://doi. org/10.13140/2.1, 2044.
								x	review	Espínola, C. F., & Torres, B. J. A. (2019). Relación entre motivación e inteligencia emocional en Educación Física: una revisión sistemática. Retos: nuevas tendencias en educación física, deporte y recreación, (36), 584-589.
							x		book chapter	Sage, L. (2017). Motivated Attention in the Multicultural Classroom. In Paradoxes in Education (pp. 69-84). Brill.
						x			no, no 3x2	Suárez-Valenzuela, S., & Suárez Riveiro, J. M. (2022). Academic goals, parenting styles, and their relationship to learning strategies in

										compulsory secondary education. Electronic Journal of Research in Educational Psychology, 20(56).
				x					yes	GEZER, M., & ŞAHİN, İ. F. (2016). Sosyal bilgiler odaklı başarı yönelimleri ölçeği (sobyö): geçerlik ve güvenirlik çalışması. Journal of Measurement and Evaluation in Education and Psychology, 7(2), 335-354.
								x	no data	Никитская, М. Г. (2019). Исследования целей достижения и направленности в контексте учебной мотивации. Современная зарубежная психология, 8(2), 26-35.
						x			no, no 3x2	Pereira, P., Santos, F., & Marinho, D. A. (2022). Portuguese Students' Perceptions About the Motivational Climate in Physical Education. Journal of Teaching in Physical Education, 1(aop), 1-10.
						x			no, no 3x2	Bardach, L., Lüftenegger, M., Oczlon, S., Spiel, C., & Schober, B. (2020). Context-related problems and university students' dropout intentions—the buffering effect of personal best goals. European Journal of Psychology of Education, 35(2), 477-493.
						x			no, no 3x2	León-del-Barco, B., Mendo-Lázaro, S., Iglesias Gallego, S., Polo-del-Río, M. I., & Iglesias Gallego, D. (2020). Academic goals and parental control in primary school children. International Journal of Environmental Research and Public Health, 17(1), 206.
							x		no, not study	Park, S., & Matsuda, N. (2018, May). Predicting students' unproductive failure on intelligent tutors in adaptive online courseware. In Proceedings of the Sixth Annual GIFT Users Symposium (Vol. 6, p. 131). US Army Research Laboratory.
							x		no, book	Tempelaar, D. (2023). Incorporating Time in Dispositional Learning Analytics Models. In Open and Inclusive Educational Practice in the Digital World (pp. 29-45). Springer, Cham.
	Achievement goal model and sport	2011-2013						x	no, not emprirical	Vansteenkiste, M., Lens, W., Elliot, A. J., Soenens, B., Mouratidis, A., & Vansteenkiste, M. (2014). Moving the achievement goal approach one step forward: Towards a systematic examination of the reasons underlying achievement goals. *Educational Psychologist*, *49*, 153-174.
				x					yes	Alasqah, S. S. (2022). Goal Orientation and Its Impact on University Students' Academic Achievement During the COVID-19 Pandemic. *SAGE Open*, *12*(2), 21582440221093617.
EBSCO	3x2 achievement goal model	anytime	relevance							
					x				dupe	Thomas, C. L. (2022). Predicting Test Anxiety Using the 3x2 Achievement Goal Model. International Journal of School & Educational Psychology, 10(2), 232–242.
					x				dupe	Thomas, C. L. (2021). Predicting test anxiety using the 3x2 achievement goal model. International Journal of School & Educational Psychology. https://doi-org.lib-e2.lib.ttu.edu/10.1080/21683603.2020.1816237

					x				dupe	Lower, L. M., Newman, T. J., & Pollard, W. S. (2016). Examination of the 3x2 Achievement Goal Model in Recreational Sport: Associations with Perceived Benefits of Sport Participation. International Journal of Sport Management, Recreation & Tourism, 26, 44–53.
					x				dupe, spanish	Méndez-Giménez, A., Cecchini-Estrada, J.-A., & Fernández-Río, J. (2014). Examinando el modelo de metas de logro 3x2 en el contexto de la Educación Física. / Examining the 3x2 Achievement Goal Model in the Physical Education context. Cuadernos de Psicología Del Deporte, 14(3), 157–167.
					x				dupe	Üztemur, S. (2020). Achievement Goals and Learning Approaches in the Context of Social Studies Teaching: Comparative Analysis of 3x2 and 2x2 Models. Participatory Educational Research, 7(2), 1–18.
	3x2 achievement goal*				x				dupe	Thomas, C. L. (2022). Predicting Test Anxiety Using the 3x2 Achievement Goal Model. International Journal of School & Educational Psychology, 10(2), 232–242.
					x				dupe	Cecchini, J.-A., Méndez-Giménez, A., & García-Romero, C. (2021). Intra-individual changes in 3x2 achievement goals, friendship goals, motivational regulations and consequences in physical education. Revista Latinoamericana de Psicología, 53.
					x				dupe	Méndez-Giménez, A., García-Romero, C., & Cecchini-Estrada, J. A. (2018). 3x2 ACHIEVEMENT GOALS, FRIENDSHIP AND AFFECTIVITY IN PHYSICAL EDUCATION: AGE-GENDER DIFFERENCES. / METAS DE LOGRO 3x2, AMISTAD Y AFECTO EN EDUCACIÓN FÍSICA: DIFERENCIAS EDAD-SEXO. Revista Internacional de Medicina y Ciencias de La Actividad Física y Del Deporte, 18(72), 637–653
					x				dupe	Thomas, C. L. (2021). Predicting test anxiety using the 3x2 achievement goal model. International Journal of School & Educational Psychology. https://doi-org.lib-e2.lib.ttu.edu/10.1080/21683603.2020.1816237
					x				dupe	Lower, L. M., Newman, T. J., & Pollard, W. S. (2016). Examination of the 3x2 Achievement Goal Model in Recreational Sport: Associations with Perceived Benefits of Sport Participation. International Journal of Sport Management, Recreation & Tourism, 26, 44–53.
				x					yes	Romero, C. G. (2021). Estudio longitudinal y transversal de metas de logro 3x2 y autodeterminación en el contexto de la educación física. / Longitudinal and Cross-Sectional Study of 3x2 Achievement Goals and Self-Determination in the Physical Education Setting. Apunts: Educacion Fisica y Deportes, 144, 81–84.
				x					yes	Méndez-Giménez, A., Cecchini, J. A., & García-Romero, C. (2018). Metas de Logro 3x2, Inteligencia Emocional y Relaciones Sociales en el Contexto de la Educación Física = 3x2 achievement goals, emotional intelligence and social relationship in the context of physical education. Revista

										Iberoamericana de Diagnóstico y Evaluación Psicologica, 49(4), 131–141. https://doi-org.lib-e2.lib.ttu.edu/10.21865/RIDEP49.4.11
					x				dupe	Méndez-Giménez, A., Cecchini-Estrada, J.-A., & Fernández-Río, J. (2014). Examinando el modelo de metas de logro 3x2 en el contexto de la Educación Física. / Examining the 3x2 Achievement Goal Model in the Physical Education context. Cuadernos de Psicología Del Deporte, 14(3), 157–167.
				x					yes	Méndez-Giménez, A., Cecchini, J.-A., Méndez-Alonso, D., Prieto, J.-A., & Fernández-Rio, J. (2018). Effect of 3x2 achievement goals and classroom goal structures on self-determined motivation: A multilevel analysis in secondary education. Anales de Psicología, 34(1), 52–62. https://doi-org.lib-e2.lib.ttu.edu/10.6018/analesps.34.1.262131
					x				dupe	García Romero, C., Méndez-Giménez, A., & Antonio Cecchini-Estrada, J. (2021). Longitudinal and Cross-Sectional Study of 3x2 Achievement Goals and Self-Determination in the Physical Education Setting. Apunts: Educació Física i Esports, 144, 83.
					x				dupe	ØVRETVEIT, K., SÆTHER, S. A., & MEHUS, I. (2019). Mastery goals are associated with training effort in Brazilian jiu-jitsu. Journal of Physical Education & Sport, 19, 1294–1299.
					x				dupe	Üztemur, S. (2020). Achievement Goals and Learning Approaches in the Context of Social Studies Teaching: Comparative Analysis of 3x2 and 2x2 Models. Participatory Educational Research, 7(2), 1–18.
				x					yes	Karahan, B. Ü. (2018). Examining the Relationship between the Achievement Goals and Teacher Engagement of Turkish Teachers. Journal of Education and Training Studies, 6(3), 101–107.
				x					yes	Zhou, M., Teo, T., & Hoi, C. K. W. (2022). Validation of a simplified Chinese version of the 3 × 2 Achievement Goal Questionnaire (AGQ-S). Journal of General Psychology, 149(1), 116–137. https://doi-org.lib-e2.lib.ttu.edu/10.1080/00221309.2020.1803194
				x					yes	Gillet, N., Lafrenière, M.-A., Huyghebaert, T., & Fouquereau, E. (2015). Autonomous and controlled reasons underlying achievement goals: Implications for the 3 × 2 achievement goal model in educational and work settings. Motivation & Emotion, 39(6), 858–875. https://doi-org.lib-e2.lib.ttu.edu/10.1007/s11031-015-9505-y
	hand-search Gillet references					x			no	Bonneville-Roussy, A., Lavigne, G. L., & Vallerand, R. J. (2011). When passion leads to excellence: The case of musicians. Psychology of Music, 39(1), 123–138. https://doi-org.lib-e2.lib.ttu.edu/10.1177/0305735609352441
						x			no	Hill, A., Tan, A.-G., & Kikuchi, A. (2008). International high school students' perceived creativity self-efficacy. Korean Journal of Thinking & Problem Solving, 18(1), 105–115.
						x			no	Ehrlich, C., & Bipp, T. (2016). Goals and subjective well-being: Further evidence for goal-striving reasons as an additional level of goal analysis.

										Personality and Individual Differences, 89, 92–99. https://doi-org.lib-e2.lib.ttu.edu/10.1016/j.paid.2015.10.001
						x			no	Gaudreau, P. (2012). Goal self-concordance moderates the relationship between achievement goals and indicators of academic adjustment. Learning and Individual Differences, 22(6), 827–832. https://doi-org.lib-e2.lib.ttu.edu/10.1016/j.lindif.2012.06.006
	knew first article			x					hand picked	Elliot, A. J., & Kou, M. Pekrun Reinhard,(2011).“A 3x2 achievement goal model”. Journal of Educational Psychology, 1033, 632-648.
EBSCO	AGQ-S	2011-2023				x			2x2	Jang, Hyun Sung. (n.d.). Investigating mastery-avoidance goals using the achievement goal questionnaire for sports (agq-s): A meta-analytic confirmatory factor analysis (ma-cfa). Current Psychology : Research & Reviews. https://doi.org/10.1007/s12144-022-03768-7
					x				dupe	Zhou, M., Teo, T., & Hoi, C. K. W. (2022). Validation of a simplified Chinese version of the 3× 2 Achievement Goal Questionnaire (AGQ-S). The Journal of General Psychology, 149(1), 116-137.
				x					yes	Danthony, S., Mascret, N., & Cury, F. (2021). The relationships between the 3× 2 achievement goal model and test anxiety in Physical Education. European Physical Education Review, 27(3), 559-573.
				x					yes	Kovács, K., Gyömbér, N., Kelemen, Á., & Fodorné Földi, R. (2019). Az észlelt autonómiatámogatás hatása a teljesítéscélokra karate utánpótláskorosztályoknál. Magyar Pszichológiai Szemle, 74(2), 163-180.
					x				dupe	Øvretveit, K., & Mehus, I. (2019). Mastery goals are associated with training effort in Brazilian jiu-jitsu. Journal of Physical Education and Sport, 19, 1294-1299.
						x			2x2	Bono, B., & Livi, S. (2016). Motivazione al successo in atleti di élite: applicazione del 2X2 Achievement Goal Framework nel nuoto. Rassegna di Psicologia.
						x			2x2	Fernandez-Rio, J., Cecchini Estrada, J. A., Mendez-Gimenez, A., Fernandez-Garcia, B., & Saavedra, P. (2014). 2× 2 Dominant achievement goal profiles in high-level swimmers. European Journal of Sport Science, 14(3), 265-272.
						x			2x2	Duff-Riddell, C., & Louw, J. (2011). Achievement goal profiles, trait-anxiety and state-emotion of young female competitive horse riders. South African Journal for Research in Sport, Physical Education and Recreation, 33(3), 37-49.
				x					yes	Mascret, N., Elliot, A. J., & Cury, F. (2015). Extending the 3× 2 achievement goal model to the sport domain: The 3× 2 Achievement Goal Questionnaire for Sport. Psychology of Sport and Exercise, 17, 7-14.
			total	46	25	27	28	7		
M.L. and C.S. hand searched									short citation	Long citation

									García-Romero et al., 2015	García-Romero, C. Relationship between the 3x2 achievement goals and perceived competence in Physical Education students Relación entre las metas de logro 3x2 y la competencia percibida en los estudiantes de Educación Física.
									Ireri et al., 2021	Anthony, M., Cecilia, N., Elizabeth, W., & Stephen, K. (2020). Achievement goal orientations as predictors of academic achievement among secondary school students in embu county, kenya. J Sch Cogn Psychol. DOI, 10, 2329-8901.
									Kilicoglu, 2019	Kılıçoğlu, G. (2019). A Study on the Relationship between Achievement Goal Orientations of Secondary School Students to Social Studies and Self-Regulation Strategies They Use. *Journal of Education and E-Learning Research*, *6*(1), 38-44.
									Mascret et al., 2022	Mascret, N., Vors, O., Marqueste, T., & Cury, F. (2022). Stress Responses, Competition, and Free-Throw Performance: The Predicting Role of Other-Approach Goals. *Psychological Reports*, *125*(6), 3049-3068.
									Méndez-Giménez et al., 2017 B	Giménez, A. M., Estrada, J. A. C., Río, J. F., Alonso, D. M., & Saborit, J. A. P. (2017). 3x2 Achievement Goals, Self-Determined Motivation and Life Satisfaction in Secondary Education. *Revista de psicodidáctica*, *22*(2), 5.
									Rivera Pérez et al., 2021 A	Rivera-Pérez, S., Fernandez-Rio, J., & Iglesias Gallego, D. (2021). Effects of an 8-week cooperative learning intervention on physical education students’ task and self-approach goals, and emotional intelligence. *International Journal of Environmental Research and Public Health*, *18*(1), 61.
									Rivera Pérez et al., 2021 B	Rivera-Pérez, S., Fernandez-Rio, J., & Gallego, D. I. (2021). Uncovering the nexus between cooperative learning contexts and achievement goals in physical education. *Perceptual and Motor Skills*, *128*(4), 1821-1835.
									Rivera Pérez et al., 2021 C	Pérez, S. R., del Barco, B. L., Bernal, J. J. G., & Gallego, D. I. (2021). Cooperative learning and approach goals in physical education: The discriminant role of individual accountability. *Revista de Psicodidáctica (English Ed.)*, *26*(1), 78-85.
									Sari et al., 2019	Sari, N. E. P., Sugiyo, S., & Sunawan, S. (2019). Achievement Goal and Homework Behavior: Mediator Effects of Achievement Emotion. *Jurnal Bimbingan Konseling*, *8*(3), 56-64.
									Yang & Cao, 2013	Yang, Y., & Cao, L. (2013). Differential influences of achievement approach goals and intrinsic/extrinsic motivation on help-seeking in e-learning. *Knowledge Management & E-Learning*, *5*(2), 153.
Wu, 2022 references									Cowden et al., 2021	Cowden, R. G., Mascret, N., & Duckett, T. R. (2021). A person-centered approach to achievement goal orientations in competitive tennis players: Associations with motivation and mental toughness. *Journal of sport and health science*, *10*(1), 73-81.

									Diseth et al., 2015	Diseth, Å. (2015). The advantages of task-based and other-based achievement goals as standards of competence. *International Journal of Educational Research*, *72*, 59-69.
									Ning, 2018	Ning, H. K. (2018). Psychometric properties of the 3× 2 Achievement Goal Questionnaire in a Hong Kong sample. *Journal of Psychoeducational Assessment*, *36*(3), 261-272.
									Mascret et al., 2015 B	Mascret, N., Elliot, A. J., & Cury, F. (2015). The 3× 2 achievement goal questionnaire for teachers. *Educational psychology*, *37*(3), 346-361.
									Shen et al., 2020	Shen, L., Lee, J., Chen, C., & Zhang, T. (2020). High school adolescents' physical activity and physical fitness: A 3× 2 achievement goal approach. *Sustainability*, *12*(15), 6005.
									Van Yperen, 2022	Van Yperen, N. W. (2022). In the context of a sports match, the goal to win is most important, right? Suggestive evidence for a hierarchical achievement goal system. *Psychology of Sport and Exercise*, *60*, 102134.
									Wang et al., 2017	Wang, C. J., Liu, W. C., Sun, Y., & Chua, L. L. (2017). Psychometric properties of the 3× 2 achievement goal questionnaire for sport. *International Journal of Sport and Exercise Psychology*, *15*(5), 460-474.
									Wei et al., 2020	Wei, C. L., Chen, W. J., Lee, M. T. S., & Tien-Liu, T. K. (2020). Psychological trends in the achievement goals of college and university athletes. *Journal of Advanced Computational Intelligence and Intelligent Informatics*, *24*(4), 468-476.
									Chung-Chin, 2014	Chung-Chin, W. (2014). Verifying the invariance of a measurement model for achievement goals theory by using the multiple group structural equation modeling. *Journal of Research in Education Sciences*, *59*(3), 59.
									Liu & Liu, 2020	Liu, S., & Liu, M. (2020). The impact of learner metacognition and goal orientation on problem-solving in a serious game environment. *Computers in Human Behavior*, *102*, 151-165.
Z.K. hand search									Ağbuğa 2014	Ağbuğa, B. Validity and reliability of 3 x 2 achievement goal model scale in turkish undergraduate students. Hacettepe Journal of Sport Sciences 2014, 25(3), 109-117.
									Çetin & Eren 2022	Çetin, G.; Eren, A. Pre-service teachers' achievement goal orientations, teacher identity, and sense of personal responsibility: The moderated mediating effects of emotions about teaching. Educational Research for Policy and Practice 2022, 21(2), 245-283.
									Kadıoglu-Akbulut & Uzuntiryaki-Kondakcı	Kadioğlu-Akbulut, C.; Uzuntiryaki-Kondakçi, E. Turkish adaptation of the 3 x 2 goal orientation scale. Bartın University Journal of Faculty of Education 2019, 8(3), 839-866.

Supplement File. Correlates entered for each study.

Study name	Subgroup within study	r	N	SE	Correlate Category	Specifics	Valance	Goal Definition
Alasqah 2022	All goals	0.15	149	0.08	Performance	Academic	Blank	All Goals
Cecchini et al. 2021	OAP1M	0.30	334	0.05	Motivation	Intrinsic Motivation	Approach	OAP
Cecchini et al. 2021	OAV1M	0.20	334	0.05	Motivation	Intrinsic Motivation	Avoidance	OAV
Cecchini et al. 2021	SAP1M	0.53	334	0.04	Motivation	Intrinsic Motivation	Approach	SAP
Cecchini et al. 2021	SAV1M	0.34	334	0.05	Motivation	Intrinsic Motivation	Avoidance	SAV
Cecchini et al. 2021	TAP1M	0.51	334	0.04	Motivation	Intrinsic Motivation	Approach	TAP
Cecchini et al. 2021	TAV1M	0.35	334	0.05	Motivation	Intrinsic Motivation	Avoidance	TAV
Cecchini et al. 2021	OAP2M	0.13	334	0.05	Motivation (Ext)	External Regulation	Approach	OAP
Cecchini et al. 2021	OAV2M	0.14	334	0.05	Motivation (Ext)	External Regulation	Avoidance	OAV
Cecchini et al. 2021	SAP2M	-0.03	334	0.05	Motivation (Ext)	External Regulation	Approach	SAP
Cecchini et al. 2021	SAV2M	0.01	334	0.05	Motivation (Ext)	External Regulation	Avoidance	SAV
Cecchini et al. 2021	TAP2M	-0.07	334	0.05	Motivation (Ext)	External Regulation	Approach	TAP
Cecchini et al. 2021	TAV2M	0.06	334	0.05	Motivation (Ext)	External Regulation	Avoidance	TAV
Cecchini et al. 2021	OAP1	0.09	334	0.05	Positive Emotions	Satisfaction w Life	Approach	OAP
Cecchini et al. 2021	OAP2	0.11	334	0.05	Positive Emotions	Satisfaction w Life	Approach	OAP
Cecchini et al. 2021	OAV1	0.06	334	0.05	Positive Emotions	Satisfaction w Life	Avoidance	OAV
Cecchini et al. 2021	OAV2	0.07	334	0.05	Positive Emotions	Satisfaction w Life	Avoidance	OAV
Cecchini et al. 2021	SAP1	0.30	334	0.05	Positive Emotions	Satisfaction w Life	Approach	SAP
Cecchini et al. 2021	SAP2	0.27	334	0.05	Positive Emotions	Satisfaction w Life	Approach	SAP
Cecchini et al. 2021	SAV1	0.21	334	0.05	Positive Emotions	Satisfaction w Life	Avoidance	SAV
Cecchini et al. 2021	SAV2	0.15	334	0.05	Positive Emotions	Satisfaction w Life	Avoidance	SAV
Cecchini et al. 2021	TAP1	0.29	334	0.05	Positive Emotions	Satisfaction w Life	Approach	TAP
Cecchini et al. 2021	TAP2	0.27	334	0.05	Positive Emotions	Satisfaction w Life	Approach	TAP
Cecchini et al. 2021	TAV1	0.24	334	0.05	Positive Emotions	Satisfaction w Life	Avoidance	TAV
Cecchini et al. 2021	TAV2	0.15	334	0.05	Positive Emotions	Satisfaction w Life	Avoidance	TAV
Cowden et al. 2021	OAP1M	0.42	323	0.05	Motivation	Autonomous Motivation	Approach	OAP

Cowden et al. 2021	OAP2M	0.27	323	0.05	Motivation	Controlled Motivation	Approach	OAP
Cowden et al. 2021	OAV1M	0.41	323	0.05	Motivation	Autonomous Motivation	Avoidance	OAV
Cowden et al. 2021	OAV2M	0.31	323	0.05	Motivation	Controlled Motivation	Avoidance	OAV
Cowden et al. 2021	SAP1M	0.45	323	0.04	Motivation	Autonomous Motivation	Approach	SAP
Cowden et al. 2021	SAP2M	0.19	323	0.05	Motivation	Controlled Motivation	Approach	SAP
Cowden et al. 2021	SAV1M	0.43	323	0.05	Motivation	Autonomous Motivation	Avoidance	SAV
Cowden et al. 2021	SAV2M	0.23	323	0.05	Motivation	Controlled Motivation	Avoidance	SAV
Cowden et al. 2021	TAP1M	0.47	323	0.04	Motivation	Autonomous Motivation	Approach	TAP
Cowden et al. 2021	TAP2M	0.21	323	0.05	Motivation	Controlled Motivation	Approach	TAP
Cowden et al. 2021	TAV1M	0.40	323	0.05	Motivation	Autonomous Motivation	Avoidance	TAV
Cowden et al. 2021	TAV2M	0.26	323	0.05	Motivation	Controlled Motivation	Avoidance	TAV
Danthony et al. 2021	OAP1	-0.07	486	0.05	Negative Emotions	Text Anxiety Worry	Approach	OAP
Danthony et al. 2021	OAP2	-0.10	486	0.05	Negative Emotions	Text Anxiety Self-Focus	Approach	OAP
Danthony et al. 2021	OAP3	0.01	486	0.05	Negative Emotions	Text Anxiety Bodily Symptoms	Approach	OAP
Danthony et al. 2021	OAP4	-0.01	486	0.05	Negative Emotions	Text Anxiety Somatic Tension	Approach	OAP
Danthony et al. 2021	OAV1	0.11	486	0.04	Negative Emotions	Text Anxiety Worry	Avoidance	OAV
Danthony et al. 2021	OAV2	0.08	486	0.05	Negative Emotions	Text Anxiety Self-Focus	Avoidance	OAV
Danthony et al. 2021	OAV3	0.09	486	0.05	Negative Emotions	Text Anxiety Bodily Symptoms	Avoidance	OAV
Danthony et al. 2021	OAV4	0.11	486	0.04	Negative Emotions	Text Anxiety Somatic Tension	Avoidance	OAV
Danthony et al. 2021	SAP1	-0.13	486	0.04	Negative Emotions	Text Anxiety Worry	Approach	SAP

Danthony et al. 2021	SAP2	-0.21	486	0.04	Negative Emotions	Text Anxiety Self-Focus	Approach	SAP
Danthony et al. 2021	SAP3	-0.14	486	0.04	Negative Emotions	Text Anxiety Bodily Symptoms	Approach	SAP
Danthony et al. 2021	SAP4	-0.19	486	0.04	Negative Emotions	Text Anxiety Somatic Tension	Approach	SAP
Danthony et al. 2021	SAV1	0.15	486	0.04	Negative Emotions	Text Anxiety Worry	Avoidance	SAV
Danthony et al. 2021	SAV2	0.07	486	0.05	Negative Emotions	Text Anxiety Self-Focus	Avoidance	SAV
Danthony et al. 2021	SAV3	0.09	486	0.05	Negative Emotions	Text Anxiety Bodily Symptoms	Avoidance	SAV
Danthony et al. 2021	SAV4	0.10	486	0.05	Negative Emotions	Text Anxiety Somatic Tension	Avoidance	SAV
Danthony et al. 2021	TAP1	-0.22	486	0.04	Negative Emotions	Text Anxiety Worry	Approach	TAP
Danthony et al. 2021	TAP2	-0.26	486	0.04	Negative Emotions	Text Anxiety Self-Focus	Approach	TAP
Danthony et al. 2021	TAP3	-0.09	486	0.05	Negative Emotions	Text Anxiety Bodily Symptoms	Approach	TAP
Danthony et al. 2021	TAP4	-0.18	486	0.04	Negative Emotions	Text Anxiety Somatic Tension	Approach	TAP
Danthony et al. 2021	TAV1	0.10	486	0.05	Negative Emotions	Text Anxiety Worry	Avoidance	TAV
Danthony et al. 2021	TAV2	0.10	486	0.05	Negative Emotions	Text Anxiety Self-Focus	Avoidance	TAV
Danthony et al. 2021	TAV3	0.03	486	0.05	Negative Emotions	Text Anxiety Bodily Symptoms	Avoidance	TAV
Danthony et al. 2021	TAV4	0.07	486	0.05	Negative Emotions	Text Anxiety Somatic Tension	Avoidance	TAV
Danthony et al. 2021	OAP5	0.30	486	0.04	Positive Emotions	Test Anxiety Perceived Control	Approach	OAP
Danthony et al. 2021	OAV5	0.02	486	0.05	Positive Emotions	Test Anxiety Perceived Control	Avoidance	OAV
Danthony et al. 2021	SAP5	0.25	486	0.04	Positive Emotions	Test Anxiety Perceived Control	Approach	SAP
Danthony et al. 2021	SAV5	-0.10	486	0.05	Positive Emotions	Test Anxiety Perceived Control	Avoidance	SAV
Danthony et al. 2021	TAP5	0.37	486	0.04	Positive Emotions	Test Anxiety Perceived Control	Approach	TAP

Danthony et al. 2021	TAV5	-0.03	486	0.05	Positive Emotions	Test Anxiety Perceived Control	Avoidance	TAV
Didin & Kasapoglu 2021	OAP1L	0.35	440	0.04	Learning	Learning Strategies in Social Studies	Approach	OAP
Didin & Kasapoglu 2021	OAV1L	0.40	440	0.04	Learning	Learning Strategies in Social Studies	Avoidance	OAV
Didin & Kasapoglu 2021	SAP1L	0.62	440	0.03	Learning	Learning Strategies in Social Studies	Approach	SAP
Didin & Kasapoglu 2021	SAV1L	0.48	440	0.04	Learning	Learning Strategies in Social Studies	Avoidance	SAV
Didin & Kasapoglu 2021	TAP1L	0.52	440	0.03	Learning	Learning Strategies in Social Studies	Approach	TAP
Didin & Kasapoglu 2021	TAV1L	0.52	440	0.03	Learning	Learning Strategies in Social Studies	Avoidance	TAV
Diseth 2015	OAP1L	0.16	217	0.07	Learning	Deep	Approach	OAP
Diseth 2015	OAP2L	0.14	217	0.07	Learning	Strategic	Approach	OAP
Diseth 2015	OAP3L	0.00	217	0.07	Learning	Surface	Approach	OAP
Diseth 2015	OAV1L	0.11	217	0.07	Learning	Deep	Avoidance	OAV
Diseth 2015	OAV2L	0.07	217	0.07	Learning	Strategic	Avoidance	OAV
Diseth 2015	OAV3L	0.12	217	0.07	Learning	Surface	Avoidance	OAV
Diseth 2015	SAP1L	0.02	217	0.07	Learning	Deep	Approach	SAP
Diseth 2015	SAP2L	-0.18	217	0.07	Learning	Strategic	Approach	SAP
Diseth 2015	SAP3L	0.25	217	0.06	Learning	Surface	Approach	SAP
Diseth 2015	SAV1L	-0.02	217	0.07	Learning	Deep	Avoidance	SAV
Diseth 2015	SAV2L	-0.13	217	0.07	Learning	Strategic	Avoidance	SAV
Diseth 2015	SAV3L	0.24	217	0.06	Learning	Surface	Avoidance	SAV
Diseth 2015	TAP1L	0.15	217	0.07	Learning	Deep	Approach	TAP
Diseth 2015	TAP2L	0.28	217	0.06	Learning	Strategic	Approach	TAP
Diseth 2015	TAP3L	0.09	217	0.07	Learning	Surface	Approach	TAP
Diseth 2015	TAV1L	-0.01	217	0.07	Learning	Deep	Avoidance	TAV
Diseth 2015	TAV2L	-0.05	217	0.07	Learning	Strategic	Avoidance	TAV
Diseth 2015	TAV3L	0.18	217	0.07	Learning	Surface	Avoidance	TAV
Diseth 2015	OAP1M	0.14	217	0.07	Motivation	Motive for Success	Approach	OAP

Diseth 2015	OAP3M	0.29	217	0.06	Motivation	Self-efficacy	Approach	OAP
Diseth 2015	OAP4M	0.08	217	0.07	Motivation	Task Value	Approach	OAP
Diseth 2015	OAV1M	0.02	217	0.07	Motivation	Motive for Success	Avoidance	OAV
Diseth 2015	OAV3M	0.11	217	0.07	Motivation	Self-efficacy	Avoidance	OAV
Diseth 2015	OAV4M	0.09	217	0.07	Motivation	Task Value	Avoidance	OAV
Diseth 2015	SAP1M	-0.05	217	0.07	Motivation	Motive for Success	Approach	SAP
Diseth 2015	SAP3M	0.01	217	0.07	Motivation	Self-efficacy	Approach	SAP
Diseth 2015	SAP4M	0.14	217	0.07	Motivation	Task Value	Approach	SAP
Diseth 2015	SAV1M	-0.04	217	0.07	Motivation	Motive for Success	Avoidance	SAV
Diseth 2015	SAV3M	-0.13	217	0.07	Motivation	Self-efficacy	Avoidance	SAV
Diseth 2015	SAV4M	0.05	217	0.07	Motivation	Task Value	Avoidance	SAV
Diseth 2015	TAP1M	0.18	217	0.07	Motivation	Motive for Success	Approach	TAP
Diseth 2015	TAP3M	0.21	217	0.07	Motivation	Self-efficacy	Approach	TAP
Diseth 2015	TAP4M	0.25	217	0.06	Motivation	Task Value	Approach	TAP
Diseth 2015	TAV1M	-0.01	217	0.07	Motivation	Motive for Success	Avoidance	TAV
Diseth 2015	TAV3M	-0.10	217	0.07	Motivation	Self-efficacy	Avoidance	TAV
Diseth 2015	TAV4M	0.08	217	0.07	Motivation	Task Value	Avoidance	TAV
Diseth 2015	OAP2M	0.06	217	0.07	Motivation (D)	Motive to Avoid Failure	Approach	OAP
Diseth 2015	OAV2M	0.12	217	0.07	Motivation (D)	Motive to Avoid Failure	Avoidance	OAV
Diseth 2015	SAP2M	0.11	217	0.07	Motivation (D)	Motive to Avoid Failure	Approach	SAP
Diseth 2015	SAV2M	0.15	217	0.07	Motivation (D)	Motive to Avoid Failure	Avoidance	SAV
Diseth 2015	TAP2M	-0.12	217	0.07	Motivation (D)	Motive to Avoid Failure	Approach	TAP
Diseth 2015	TAV2M	0.14	217	0.07	Motivation (D)	Motive to Avoid Failure	Avoidance	TAV
Diseth 2015	OAP1P	0.26	217	0.06	Performance	Academic Achievement	Approach	OAP
Diseth 2015	OAV1P	0.02	217	0.07	Performance	Academic Achievement	Avoidance	OAV

Diseth 2015	SAP1P	-0.23	217	0.06	Performance	Academic Achievement	Approach	SAP
Diseth 2015	SAV1P	-0.02	217	0.07	Performance	Academic Achievement	Avoidance	SAV
Diseth 2015	TAP1P	0.12	217	0.07	Performance	Academic Achievement	Approach	TAP
Diseth 2015	TAV1P	0.05	217	0.07	Performance	Academic Achievement	Avoidance	TAV
Garcia-Romero 2015	OAP1M	0.33	205	0.06	Motivation	Competence Needs	Approach	OAP
Garcia-Romero 2015	OAV1M	0.28	205	0.06	Motivation	Competence Needs	Avoidance	OAV
Garcia-Romero 2015	SAP1M	0.54	205	0.05	Motivation	Competence Needs	Approach	SAP
Garcia-Romero 2015	SAV1M	0.36	205	0.06	Motivation	Competence Needs	Avoidance	SAV
Garcia-Romero 2015	TAP1M	0.61	205	0.04	Motivation	Competence Needs	Approach	TAP
Garcia-Romero 2015	TAV1M	0.36	205	0.06	Motivation	Competence Needs	Avoidance	TAV
García-Romero et al. 2020	OAP1M	0.32	1706	0.02	Motivation	Autonomy Need Satisfaction	Approach	OAP
García-Romero et al. 2020	OAV1M	0.31	1706	0.02	Motivation	Autonomy Need Satisfaction	Avoidance	OAV
García-Romero et al. 2020	SAP1M	0.43	1706	0.02	Motivation	Autonomy Need Satisfaction	Approach	SAP
García-Romero et al. 2020	SAV1M	0.33	1706	0.02	Motivation	Autonomy Need Satisfaction	Avoidance	SAV
García-Romero et al. 2020	TAP1M	0.44	1706	0.02	Motivation	Autonomy Need Satisfaction	Approach	TAP
García-Romero et al. 2020	TAV1M	0.30	1706	0.02	Motivation	Autonomy Need Satisfaction	Avoidance	TAV
García-Romero et al. 2022	OAP1M	0.32	1706	0.02	Motivation	Autonomy	Approach	OAP
García-Romero et al. 2022	OAP2M	0.39	1706	0.02	Motivation	Competence	Approach	OAP
García-Romero et al. 2022	OAP3M	0.22	1706	0.02	Motivation	Relatedness	Approach	OAP
García-Romero et al. 2022	OAP4M	-0.02	1706	0.02	Motivation	SDI	Approach	OAP
García-Romero et al. 2022	OAV1M	0.31	1706	0.02	Motivation	Autonomy	Avoidance	OAV
García-Romero et al. 2022	OAV2M	0.38	1706	0.02	Motivation	Competence	Avoidance	OAV
García-Romero et al. 2022	OAV3M	0.24	1706	0.02	Motivation	Relatedness	Avoidance	OAV
García-Romero et al. 2022	OAV4M	0.07	1706	0.02	Motivation	SDI	Avoidance	OAV

García-Romero et al. 2022	SAP1M	0.43	1706	0.02	Motivation	Autonomy	Approach	SAP
García-Romero et al. 2022	SAP2M	0.55	1706	0.02	Motivation	Competence	Approach	SAP
García-Romero et al. 2022	SAP3M	0.43	1706	0.02	Motivation	Relatedness	Approach	SAP
García-Romero et al. 2022	SAP4M	0.36	1706	0.02	Motivation	SDI	Approach	SAP
García-Romero et al. 2022	SAV1M	0.33	1706	0.02	Motivation	Autonomy	Avoidance	SAV
García-Romero et al. 2022	SAV2M	0.42	1706	0.02	Motivation	Competence	Avoidance	SAV
García-Romero et al. 2022	SAV3M	0.29	1706	0.02	Motivation	Relatedness	Avoidance	SAV
García-Romero et al. 2022	SAV4M	0.26	1706	0.02	Motivation	SDI	Avoidance	SAV
García-Romero et al. 2022	TAP1M	0.44	1706	0.02	Motivation	Autonomy	Approach	TAP
García-Romero et al. 2022	TAP2M	0.61	1706	0.02	Motivation	Competence	Approach	TAP
García-Romero et al. 2022	TAP3M	0.46	1706	0.02	Motivation	Relatedness	Approach	TAP
García-Romero et al. 2022	TAP4M	0.39	1706	0.02	Motivation	SDI	Approach	TAP
García-Romero et al. 2022	TAV1M	0.30	1706	0.02	Motivation	Autonomy	Avoidance	TAV
García-Romero et al. 2022	TAV2M	0.40	1706	0.02	Motivation	Competence	Avoidance	TAV
García-Romero et al. 2022	TAV3M	0.32	1706	0.02	Motivation	Relatedness	Avoidance	TAV
García-Romero et al. 2022	TAV4M	0.32	1706	0.02	Motivation	SDI	Avoidance	TAV
García-Romero et al. 2022	OAP1	0.20	1706	0.02	Positive Emotions	Satisfaction w Life	Approach	OAP
García-Romero et al. 2022	OAV1	0.20	1706	0.02	Positive Emotions	Satisfaction w Life	Avoidance	OAV
García-Romero et al. 2022	SAP1	0.31	1706	0.02	Positive Emotions	Satisfaction w Life	Approach	SAP
García-Romero et al. 2022	SAV1	0.25	1706	0.02	Positive Emotions	Satisfaction w Life	Avoidance	SAV
García-Romero et al. 2022	TAP1	0.37	1706	0.02	Positive Emotions	Satisfaction w Life	Approach	TAP
García-Romero et al. 2022	TAV1	0.29	1706	0.02	Positive Emotions	Satisfaction w Life	Avoidance	TAV
Gillet et al. 2015 S1	OAP1L	0.07	278	0.06	Learning	Study Engagement	Approach	OAP
Gillet et al. 2015 S1	OAV1L	0.01	278	0.06	Learning	Study Engagement	Avoidance	OAV
Gillet et al. 2015 S1	SAP1L	0.13	278	0.06	Learning	Study Engagement	Approach	SAP
Gillet et al. 2015 S1	SAV1L	0.03	278	0.06	Learning	Study Engagement	Avoidance	SAV
Gillet et al. 2015 S1	TAP1L	0.13	278	0.06	Learning	Study Engagement	Approach	TAP
Gillet et al. 2015 S1	TAV1L	0.02	278	0.06	Learning	Study Engagement	Avoidance	TAV
Gillet et al. 2015 S1	OAP1	0.07	278	0.06	Positive Emotions	Satisfaction	Approach	OAP
Gillet et al. 2015 S1	OAP2	0.12	278	0.06	Positive Emotions	Positive Affect	Approach	OAP
Gillet et al. 2015 S1	OAV1	-0.08	278	0.06	Positive Emotions	Satisfaction	Avoidance	OAV

Gillet et al. 2015 S1	OAV2	0.01	278	0.06	Positive Emotions	Positive Affect	Avoidance	OAV
Gillet et al. 2015 S1	SAP1	0.13	278	0.06	Positive Emotions	Satisfaction	Approach	SAP
Gillet et al. 2015 S1	SAP2	0.11	278	0.06	Positive Emotions	Positive Affect	Approach	SAP
Gillet et al. 2015 S1	SAV1	0.01	278	0.06	Positive Emotions	Satisfaction	Avoidance	SAV
Gillet et al. 2015 S1	SAV2	0.00	278	0.06	Positive Emotions	Positive Affect	Avoidance	SAV
Gillet et al. 2015 S1	TAP1	0.14	278	0.06	Positive Emotions	Satisfaction	Approach	TAP
Gillet et al. 2015 S1	TAP2	0.14	278	0.06	Positive Emotions	Positive Affect	Approach	TAP
Gillet et al. 2015 S1	TAV1	0.04	278	0.06	Positive Emotions	Satisfaction	Avoidance	TAV
Gillet et al. 2015 S1	TAV2	0.01	278	0.06	Positive Emotions	Positive Affect	Avoidance	TAV
Gillet et al. 2015 S2	OAP1L	0.11	327	0.05	Learning	Study Engagement	Approach	OAP
Gillet et al. 2015 S2	OAV1L	0.11	327	0.05	Learning	Study Engagement	Avoidance	OAV
Gillet et al. 2015 S2	SAP1L	0.11	327	0.05	Learning	Study Engagement	Approach	SAP
Gillet et al. 2015 S2	SAV1L	0.00	327	0.06	Learning	Study Engagement	Avoidance	SAV
Gillet et al. 2015 S2	TAP1L	0.17	327	0.05	Learning	Study Engagement	Approach	TAP
Gillet et al. 2015 S2	TAV1L	0.14	327	0.05	Learning	Study Engagement	Avoidance	TAV
Gillet et al. 2015 S2	OAP3	0.05	327	0.06	Negative Emotions	Anxiety	Approach	OAP
Gillet et al. 2015 S2	OAV3	0.14	327	0.05	Negative Emotions	Anxiety	Avoidance	OAV
Gillet et al. 2015 S2	SAP3	0.09	327	0.06	Negative Emotions	Anxiety	Approach	SAP
Gillet et al. 2015 S2	SAV3	0.05	327	0.06	Negative Emotions	Anxiety	Avoidance	SAV
Gillet et al. 2015 S2	TAP3	0.07	327	0.06	Negative Emotions	Anxiety	Approach	TAP
Gillet et al. 2015 S2	TAV3	0.12	327	0.05	Negative Emotions	Anxiety	Avoidance	TAV
Gillet et al. 2015 S2	OAP1	0.07	327	0.06	Positive Emotions	Satisfaction	Approach	OAP
Gillet et al. 2015 S2	OAP2	0.15	327	0.05	Positive Emotions	Positive Affect	Approach	OAP
Gillet et al. 2015 S2	OAV1	0.11	327	0.05	Positive Emotions	Satisfaction	Avoidance	OAV
Gillet et al. 2015 S2	OAV2	0.15	327	0.05	Positive Emotions	Positive Affect	Avoidance	OAV
Gillet et al. 2015 S2	SAP1	0.09	327	0.06	Positive Emotions	Satisfaction	Approach	SAP
Gillet et al. 2015 S2	SAP2	0.10	327	0.06	Positive Emotions	Positive Affect	Approach	SAP
Gillet et al. 2015 S2	SAV1	-0.01	327	0.06	Positive Emotions	Satisfaction	Avoidance	SAV
Gillet et al. 2015 S2	SAV2	0.04	327	0.06	Positive Emotions	Positive Affect	Avoidance	SAV
Gillet et al. 2015 S2	TAP1	0.19	327	0.05	Positive Emotions	Satisfaction	Approach	TAP
Gillet et al. 2015 S2	TAP2	0.21	327	0.05	Positive Emotions	Positive Affect	Approach	TAP

Gillet et al. 2015 S2	TAV1	0.18	327	0.05	Positive Emotions	Satisfaction	Avoidance	TAV
Gillet et al. 2015 S2	TAV2	0.14	327	0.05	Positive Emotions	Positive Affect	Avoidance	TAV
Gillet et al. 2015 S3	OAP1L	0.21	169	0.07	Learning	Study Engagement	Approach	OAP
Gillet et al. 2015 S3	OAV1L	-0.02	169	0.08	Learning	Study Engagement	Avoidance	OAV
Gillet et al. 2015 S3	SAP1L	0.29	169	0.07	Learning	Study Engagement	Approach	SAP
Gillet et al. 2015 S3	SAV1L	-0.06	169	0.08	Learning	Study Engagement	Avoidance	SAV
Gillet et al. 2015 S3	TAP1L	0.23	169	0.07	Learning	Study Engagement	Approach	TAP
Gillet et al. 2015 S3	TAV1L	-0.02	169	0.08	Learning	Study Engagement	Avoidance	TAV
Gillet et al. 2015 S3	OAP1	0.09	169	0.08	Positive Emotions	Satisfaction	Approach	OAP
Gillet et al. 2015 S3	OAP2	0.19	169	0.07	Positive Emotions	Positive Affect	Approach	OAP
Gillet et al. 2015 S3	OAV1	-0.02	169	0.08	Positive Emotions	Satisfaction	Avoidance	OAV
Gillet et al. 2015 S3	OAV2	0.07	169	0.08	Positive Emotions	Positive Affect	Avoidance	OAV
Gillet et al. 2015 S3	SAP1	0.11	169	0.08	Positive Emotions	Satisfaction	Approach	SAP
Gillet et al. 2015 S3	SAP2	0.28	169	0.07	Positive Emotions	Positive Affect	Approach	SAP
Gillet et al. 2015 S3	SAV1	-0.08	169	0.08	Positive Emotions	Satisfaction	Avoidance	SAV
Gillet et al. 2015 S3	SAV2	-0.05	169	0.08	Positive Emotions	Positive Affect	Avoidance	SAV
Gillet et al. 2015 S3	TAP1	0.15	169	0.08	Positive Emotions	Satisfaction	Approach	TAP
Gillet et al. 2015 S3	TAP2	0.31	169	0.07	Positive Emotions	Positive Affect	Approach	TAP
Gillet et al. 2015 S3	TAV1	-0.02	169	0.08	Positive Emotions	Satisfaction	Avoidance	TAV
Gillet et al. 2015 S3	TAV2	-0.01	169	0.08	Positive Emotions	Positive Affect	Avoidance	TAV
Hidayat et al. 2018 B	All goals2L	0.43	538	0.04	Learning	Metacognition Awareness	Blank	All Goals
Hidayat et al. 2018 B	All goals3L	0.54	538	0.03	Learning	Metacognition Cognitive Strategy	Blank	All Goals
Hidayat et al. 2018 B	All goals4L	0.48	538	0.03	Learning	Metacognition Planning	Blank	All Goals
Hidayat et al. 2018 B	All goals5L	0.48	538	0.03	Learning	Metacognition Self-checking	Blank	All Goals
Hidayat et al. 2018 B	All goals1L	0.46	538	0.03	Performance	Math Modeling Competency	Blank	All Goals
Hidayat et al. 2018 C	SAP1L	0.34	483	0.04	Learning	Metacognition	Approach	SAP
Hidayat et al. 2018 C	SAV1L	0.30	483	0.04	Learning	Metacognition	Avoidance	SAV

Hidayat et al. 2018 C	TAP1L	0.26	483	0.04	Learning	Metacognition	Approach	TAP
Hidayat et al. 2018 C	TAV1L	0.30	483	0.04	Learning	Metacognition	Avoidance	TAV
Ireri et al. 2021	Approach goals	0.20	385	0.05	Performance	Academic Achievement	Approach	OAP
Ireri et al. 2021	Avoidance goals	-0.15	385	0.05	Performance	Academic Achievement	Approach	OAP
Kiliçoglu 2019	OAP1L	0.13	346	0.05	Learning	Use of Cognitive Strategies	Approach	OAP
Kiliçoglu 2019	OAP2L	0.22	346	0.05	Learning	Self-regulation	Approach	OAP
Kiliçoglu 2019	OAV1L	0.33	346	0.05	Learning	Use of Cognitive Strategies	Avoidance	OAV
Kiliçoglu 2019	OAV2L	0.26	346	0.05	Learning	Self-regulation	Avoidance	OAV
Kiliçoglu 2019	SAP1L	0.61	346	0.03	Learning	Use of Cognitive Strategies	Approach	SAP
Kiliçoglu 2019	SAP2L	0.41	346	0.04	Learning	Self-regulation	Approach	SAP
Kiliçoglu 2019	SAV1L	0.34	346	0.05	Learning	Use of Cognitive Strategies	Avoidance	SAV
Kiliçoglu 2019	SAV2L	0.29	346	0.05	Learning	Self-regulation	Avoidance	SAV
Kiliçoglu 2019	TAP1L	0.58	346	0.04	Learning	Use of Cognitive Strategies	Approach	TAP
Kiliçoglu 2019	TAP2L	0.34	346	0.05	Learning	Self-regulation	Approach	TAP
Kiliçoglu 2019	TAV1L	0.53	346	0.04	Learning	Use of Cognitive Strategies	Avoidance	TAV
Kiliçoglu 2019	TAV2L	0.40	346	0.05	Learning	Self-regulation	Avoidance	TAV
León-del-Barco et al. 2019	OAP1L	0.14	700	0.04	Learning	Academic Consequences of Teamwork	Approach	OAP
León-del-Barco et al. 2019	OAP2L	-0.01	700	0.04	Learning	Assessment of Interactions during Teamwork	Approach	OAP
León-del-Barco et al. 2019	OAV1L	0.12	700	0.04	Learning	Academic Consequences of Teamwork	Avoidance	OAV
León-del-Barco et al. 2019	OAV2L	0.07	700	0.04	Learning	Assessment of Interactions during Teamwork	Avoidance	OAV

León-del-Barco et al. 2019	SAP1L	0.22	700	0.04	Learning	Academic Consequences of Teamwork	Approach	SAP
León-del-Barco et al. 2019	SAP2L	0.22	700	0.04	Learning	Assessment of Interactions during Teamwork	Approach	SAP
León-del-Barco et al. 2019	SAV1L	0.13	700	0.04	Learning	Academic Consequences of Teamwork	Avoidance	SAV
León-del-Barco et al. 2019	SAV2L	0.06	700	0.04	Learning	Assessment of Interactions during Teamwork	Avoidance	SAV
León-del-Barco et al. 2019	TAP1L	0.13	700	0.04	Learning	Academic Consequences of Teamwork	Approach	TAP
León-del-Barco et al. 2019	TAP2L	0.28	700	0.03	Learning	Assessment of Interactions during Teamwork	Approach	TAP
León-del-Barco et al. 2019	TAV1L	0.04	700	0.04	Learning	Academic Consequences of Teamwork	Avoidance	TAV
León-del-Barco et al. 2019	TAV2L	0.13	700	0.04	Learning	Assessment of Interactions during Teamwork	Avoidance	TAV
Liu & Liu 2020	OAP1M	0.21	159	0.08	Learning	Metacognition	Approach	OAP
Liu & Liu 2020	OAV1M	0.21	159	0.08	Learning	Metacognition	Avoidance	OAV
Liu & Liu 2020	SAP1M	0.27	159	0.07	Learning	Metacognition	Approach	SAP
Liu & Liu 2020	SAV1M	0.23	159	0.08	Learning	Metacognition	Avoidance	SAV
Liu & Liu 2020	TAP1M	0.27	159	0.07	Learning	Metacognition	Approach	TAP
Liu & Liu 2020	TAV1M	0.19	159	0.08	Learning	Metacognition	Avoidance	TAV
Lüftenegger et al. 2016	OAP2	0.01	388	0.05	Negative Emotions	Boredom	Approach	OAP
Lüftenegger et al. 2016	OAV2	0.06	388	0.05	Negative Emotions	Boredom	Avoidance	OAV
Lüftenegger et al. 2016	SAP2	-0.08	388	0.05	Negative Emotions	Boredom	Approach	SAP
Lüftenegger et al. 2016	SAV2	-0.03	388	0.05	Negative Emotions	Boredom	Avoidance	SAV
Lüftenegger et al. 2016	TAP2	-0.17	388	0.05	Negative Emotions	Boredom	Approach	TAP

Lüftenegger et al. 2016	TAV2	-0.03	388	0.05	Negative Emotions	Boredom	Avoidance	TAV
Lüftenegger et al. 2016	OAP1P	0.16	388	0.05	Performance	Academic	Approach	OAP
Lüftenegger et al. 2016	OAV1P	0.07	388	0.05	Performance	Academic	Avoidance	OAV
Lüftenegger et al. 2016	SAP1P	0.06	388	0.05	Performance	Academic	Approach	SAP
Lüftenegger et al. 2016	SAV1P	0.12	388	0.05	Performance	Academic	Avoidance	SAV
Lüftenegger et al. 2016	TAP1P	0.17	388	0.05	Performance	Academic	Approach	TAP
Lüftenegger et al. 2016	TAV1P	0.05	388	0.05	Performance	Academic	Avoidance	TAV
Lüftenegger et al. 2016	OAP1	0.25	388	0.05	Positive Emotions	Enjoyment	Approach	OAP
Lüftenegger et al. 2016	OAV1	0.17	388	0.05	Positive Emotions	Enjoyment	Avoidance	OAV
Lüftenegger et al. 2016	SAP1	0.32	388	0.05	Positive Emotions	Enjoyment	Approach	SAP
Lüftenegger et al. 2016	SAV1	0.16	388	0.05	Positive Emotions	Enjoyment	Avoidance	SAV
Lüftenegger et al. 2016	TAP1	0.17	388	0.05	Positive Emotions	Enjoyment	Approach	TAP
Lüftenegger et al. 2016	TAV1	0.04	388	0.05	Positive Emotions	Enjoyment	Avoidance	TAV
Mascret et al. 2015 A	OAP12D	0.08	302	0.06	Individual Difference	Incremental Theory	Approach	OAP
Mascret et al. 2015 A	OAP1ID	0.19	302	0.06	Individual Difference	Entity Theory	Approach	OAP
Mascret et al. 2015 A	OAV12D	0.07	302	0.06	Individual Difference	Incremental Theory	Avoidance	OAV
Mascret et al. 2015 A	OAV1ID	0.13	302	0.06	Individual Difference	Entity Theory	Avoidance	OAV
Mascret et al. 2015 A	SAP12D	0.24	302	0.05	Individual Difference	Incremental Theory	Approach	SAP
Mascret et al. 2015 A	SAP1ID	-0.03	302	0.06	Individual Difference	Entity Theory	Approach	SAP
Mascret et al. 2015 A	SAV12D	0.09	302	0.06	Individual Difference	Incremental Theory	Avoidance	SAV
Mascret et al. 2015 A	SAV1ID	-0.01	302	0.06	Individual Difference	Entity Theory	Avoidance	SAV
Mascret et al. 2015 A	TAP12D	0.23	302	0.05	Individual Difference	Incremental Theory	Approach	TAP
Mascret et al. 2015 A	TAP1ID	-0.01	302	0.06	Individual Difference	Entity Theory	Approach	TAP

Mascret et al. 2015 A	TAV12D	0.09	302	0.06	Individual Difference	Incremental Theory	Avoidance	TAV
Mascret et al. 2015 A	TAV1ID	-0.05	302	0.06	Individual Difference	Entity Theory	Avoidance	TAV
Mascret et al. 2015 A S2	OAP1M	0.00	302	0.06	Motivation	Intrinsic Interest	Approach	OAP
Mascret et al. 2015 A S2	OAV1M	-0.03	302	0.06	Motivation	Intrinsic Interest	Avoidance	OAV
Mascret et al. 2015 A S2	SAP1M	0.24	302	0.05	Motivation	Intrinsic Interest	Approach	SAP
Mascret et al. 2015 A S2	SAV1M	0.09	302	0.06	Motivation	Intrinsic Interest	Avoidance	SAV
Mascret et al. 2015 A S2	TAP1M	0.27	302	0.05	Motivation	Intrinsic Interest	Approach	TAP
Mascret et al. 2015 A S2	TAV1M	0.01	302	0.06	Motivation	Intrinsic Interest	Avoidance	TAV
Mascret et al. 2015 B	OAP12D	0.00	304	0.06	Individual Difference	Incremental Theory	Approach	OAP
Mascret et al. 2015 B	OAP1ID	0.26	304	0.05	Individual Difference	Entity Theory	Approach	OAP
Mascret et al. 2015 B	OAV12D	-0.10	304	0.06	Individual Difference	Incremental Theory	Avoidance	OAV
Mascret et al. 2015 B	OAV1ID	0.41	304	0.05	Individual Difference	Entity Theory	Avoidance	OAV
Mascret et al. 2015 B	SAP12D	0.19	304	0.06	Individual Difference	Incremental Theory	Approach	SAP
Mascret et al. 2015 B	SAP1ID	0.03	304	0.06	Individual Difference	Entity Theory	Approach	SAP
Mascret et al. 2015 B	SAV12D	0.12	304	0.06	Individual Difference	Incremental Theory	Avoidance	SAV
Mascret et al. 2015 B	SAV1ID	0.11	304	0.06	Individual Difference	Entity Theory	Avoidance	SAV
Mascret et al. 2015 B	TAP12D	0.00	304	0.06	Individual Difference	Incremental Theory	Approach	TAP
Mascret et al. 2015 B	TAP1D	-0.09	304	0.06	Individual Difference	Entity Theory	Approach	TAP
Mascret et al. 2015 B	TAV12D	0.06	304	0.06	Individual Difference	Incremental Theory	Avoidance	TAV
Mascret et al. 2015 B	TAV1ID	-0.12	304	0.06	Individual Difference	Entity Theory	Avoidance	TAV
Mascret et al. 2015 B	OAP1L	-0.06	304	0.06	Learning	Mastery Teaching Practices	Approach	OAP

Mascret et al. 2015 B	OAP2L	0.12	304	0.06	Learning	Performance Teaching Practices	Approach	OAP
Mascret et al. 2015 B	OAV1L	0.05	304	0.06	Learning	Mastery Teaching Practices	Avoidance	OAV
Mascret et al. 2015 B	OAV2L	0.13	304	0.06	Learning	Performance Teaching Practices	Avoidance	OAV
Mascret et al. 2015 B	SAP1L	0.02	304	0.06	Learning	Mastery Teaching Practices	Approach	SAP
Mascret et al. 2015 B	SAP2L	0.02	304	0.06	Learning	Performance Teaching Practices	Approach	SAP
Mascret et al. 2015 B	SAV1L	0.00	304	0.06	Learning	Mastery Teaching Practices	Avoidance	SAV
Mascret et al. 2015 B	SAV2L	0.07	304	0.06	Learning	Performance Teaching Practices	Avoidance	SAV
Mascret et al. 2015 B	TAP1L	0.11	304	0.06	Learning	Mastery Teaching Practices	Approach	TAP
Mascret et al. 2015 B	TAP2L	-0.13	304	0.06	Learning	Performance Teaching Practices	Approach	TAP
Mascret et al. 2015 B	TAV1L	0.09	304	0.06	Learning	Mastery Teaching Practices	Avoidance	TAV
Mascret et al. 2015 B	TAV2L	-0.07	304	0.06	Learning	Performance Teaching Practices	Avoidance	TAV
Mascret et al. 2015 B	OAP1M	-0.04	304	0.06	Motivation	Intrinsic Interest	Approach	OAP
Mascret et al. 2015 B	OAV1M	0.09	304	0.06	Motivation	Intrinsic Interest	Avoidance	OAV
Mascret et al. 2015 B	SAP1M	0.21	304	0.06	Motivation	Intrinsic Interest	Approach	SAP
Mascret et al. 2015 B	SAV1M	0.08	304	0.06	Motivation	Intrinsic Interest	Avoidance	SAV
Mascret et al. 2015 B	TAP1M	0.24	304	0.05	Motivation	Intrinsic Interest	Approach	TAP
Mascret et al. 2015 B	TAV1M	0.31	304	0.05	Motivation	Intrinsic Interest	Avoidance	TAV
Mascret et al. 2022	OAP1	-0.01	38	0.17	Negative Emotions	Cognitive Anxiety (pre stress)	Approach	OAP
Mascret et al. 2022	OAP2	0.14	38	0.17	Negative Emotions	Somatic Anxiety (pre stress)	Approach	OAP
Mascret et al. 2022	OAP3	-0.03	38	0.17	Negative Emotions	Cognitive Anxiety (pre stress)	Approach	OAP
Mascret et al. 2022	OAP4	0.26	38	0.16	Negative Emotions	Somatc Anxiety (pre stress)	Approach	OAP

Mascret et al. 2022	OAV1	-0.01	38	0.17	Negative Emotions	Cognitive Anxiety (post stress)	Avoidance	OAV
Mascret et al. 2022	OAV2	-0.08	38	0.17	Negative Emotions	Somatic Anxiety (post stress)	Avoidance	OAV
Mascret et al. 2022	OAV3	-0.01	38	0.17	Negative Emotions	Cognitive Anxiety (post stress)	Avoidance	OAV
Mascret et al. 2022	OAV4	0.11	38	0.17	Negative Emotions	Somatic Anxiety (post stress)	Avoidance	OAV
Mascret et al. 2022	OAP1P	0.04	38	0.17	Performance	Pre stress	Approach	OAP
Mascret et al. 2022	OAP2P	-0.10	38	0.17	Performance	Pre stress	Approach	OAP
Mascret et al. 2022	OAV1P	0.49	38	0.13	Performance	Post stress	Avoidance	OAV
Mascret et al. 2022	OAV2P	-0.02	38	0.17	Performance	Post stress	Avoidance	OAV
Méndez-Giménez et al. 2017 A	OAP1M	0.27	1347	0.03	Motivation	Intrinsic Motivation	Approach	OAP
Méndez-Giménez et al. 2017 A	OAV1M	0.31	1347	0.02	Motivation	Intrinsic Motivation	Avoidance	OAV
Méndez-Giménez et al. 2017 A	SAP1M	0.48	1347	0.02	Motivation	Intrinsic Motivation	Approach	SAP
Méndez-Giménez et al. 2017 A	SAV1M	0.38	1347	0.02	Motivation	Intrinsic Motivation	Avoidance	SAV
Méndez-Giménez et al. 2017 A	TAP1M	0.45	1347	0.02	Motivation	Intrinsic Motivation	Approach	TAP
Méndez-Giménez et al. 2017 A	TAV1M	0.40	1347	0.02	Motivation	Intrinsic Motivation	Avoidance	TAV
Méndez-Giménez et al. 2017 A	OAP2M	0.17	1347	0.03	Motivation (Ext)	External Regulation	Approach	OAP
Méndez-Giménez et al. 2017 A	OAV2M	0.18	1347	0.03	Motivation (Ext)	External Regulation	Avoidance	OAV
Méndez-Giménez et al. 2017 A	SAP2M	0.18	1347	0.03	Motivation (Ext)	External Regulation	Approach	SAP
Méndez-Giménez et al. 2017 A	SAV2M	0.23	1347	0.03	Motivation (Ext)	External Regulation	Avoidance	SAV
Méndez-Giménez et al. 2017 A	TAP2M	0.22	1347	0.03	Motivation (Ext)	External Regulation	Approach	TAP
Méndez-Giménez et al. 2017 A	TAV2M	0.23	1347	0.03	Motivation (Ext)	External Regulation	Avoidance	TAV
Méndez-Giménez et al. 2017 A	OAP2	0.15	1347	0.03	Negative Emotions	Negative Affect	Approach	OAP
Méndez-Giménez et al. 2017 A	OAV2	0.09	1347	0.03	Negative Emotions	Negative Affect	Avoidance	OAV
Méndez-Giménez et al. 2017 A	SAP2	-0.02	1347	0.03	Negative Emotions	Negative Affect	Approach	SAP
Méndez-Giménez et al. 2017 A	SAV2	0.03	1347	0.03	Negative Emotions	Negative Affect	Avoidance	SAV
Méndez-Giménez et al. 2017 A	TAP2	0.01	1347	0.03	Negative Emotions	Negative Affect	Approach	TAP
Méndez-Giménez et al. 2017 A	TAV2	0.01	1347	0.03	Negative Emotions	Negative Affect	Avoidance	TAV
Méndez-Giménez et al. 2017 A	OAP1	0.21	1347	0.03	Positive Emotions	Positive Affect	Approach	OAP
Méndez-Giménez et al. 2017 A	OAV1	0.20	1347	0.03	Positive Emotions	Positive Affect	Avoidance	OAV

Méndez-Giménez et al. 2017 A	SAP1	0.33	1347	0.02	Positive Emotions	Positive Affect	Approach	SAP
Méndez-Giménez et al. 2017 A	SAV1	0.28	1347	0.03	Positive Emotions	Positive Affect	Avoidance	SAV
Méndez-Giménez et al. 2017 A	TAP1	0.33	1347	0.02	Positive Emotions	Positive Affect	Approach	TAP
Méndez-Giménez et al. 2017 A	TAV1	0.27	1347	0.03	Positive Emotions	Positive Affect	Avoidance	TAV
Méndez-Giménez et al. 2017 B	OAP1M	0.11	2630	0.02	Motivation	SDI	Approach	OAP
Méndez-Giménez et al. 2017 B	OAV1M	0.15	2630	0.02	Motivation	SDI	Avoidance	OAV
Méndez-Giménez et al. 2017 B	SAP1M	0.53	2630	0.01	Motivation	SDI	Approach	SAP
Méndez-Giménez et al. 2017 B	SAV1M	0.32	2630	0.02	Motivation	SDI	Avoidance	SAV
Méndez-Giménez et al. 2017 B	TAP1M	0.37	2630	0.02	Motivation	SDI	Approach	TAP
Méndez-Giménez et al. 2017 B	TAV1M	0.26	2630	0.02	Motivation	SDI	Avoidance	TAV
Méndez-Giménez et al. 2017 B	OAP1	0.17	2630	0.02	Positive Emotions	Satisfaction w Life	Approach	OAP
Méndez-Giménez et al. 2017 B	OAV1	0.15	2630	0.02	Positive Emotions	Satisfaction w Life	Avoidance	OAV
Méndez-Giménez et al. 2017 B	SAP1	0.32	2630	0.02	Positive Emotions	Satisfaction w Life	Approach	SAP
Méndez-Giménez et al. 2017 B	SAV1	0.21	2630	0.02	Positive Emotions	Satisfaction w Life	Avoidance	SAV
Méndez-Giménez et al. 2017 B	TAP1	0.27	2630	0.02	Positive Emotions	Satisfaction w Life	Approach	TAP
Méndez-Giménez et al. 2017 B	TAV1	0.20	2630	0.02	Positive Emotions	Satisfaction w Life	Avoidance	TAV
Méndez-Giménez et al. 2018a	OAP1M	0.22	1689	0.02	Motivation	Relatedness Needs Satisfaction	Approach	OAP
Méndez-Giménez et al. 2018a	OAV1M	0.24	1689	0.02	Motivation	Relatedness Needs Satisfaction	Avoidance	OAV
Méndez-Giménez et al. 2018a	SAP1M	0.43	1689	0.02	Motivation	Relatedness Needs Satisfaction	Approach	SAP
Méndez-Giménez et al. 2018a	SAV1M	0.30	1689	0.02	Motivation	Relatedness Needs Satisfaction	Avoidance	SAV
Méndez-Giménez et al. 2018a	TAP1M	0.46	1689	0.02	Motivation	Relatedness Needs Satisfaction	Approach	TAP
Méndez-Giménez et al. 2018a	TAV1M	0.33	1689	0.02	Motivation	Relatedness Needs Satisfaction	Avoidance	TAV
Méndez-Giménez et al. 2018b S1	OAP2	0.13	405	0.05	Negative Emotions	Negative Affect	Approach	OAP
Méndez-Giménez et al. 2018b S1	OAV2	0.10	405	0.05	Negative Emotions	Negative Affect	Avoidance	OAV
Méndez-Giménez et al. 2018b S1	SAP2	-0.10	405	0.05	Negative Emotions	Negative Affect	Approach	SAP

Méndez-Giménez et al. 2018b S1	SAV2	-0.01	405	0.05	Negative Emotions	Negative Affect	Avoidance	SAV
Méndez-Giménez et al. 2018b S1	TAP2	0.01	405	0.05	Negative Emotions	Negative Affect	Approach	TAP
Méndez-Giménez et al. 2018b S1	TAV2	-0.02	405	0.05	Negative Emotions	Negative Affect	Avoidance	TAV
Méndez-Giménez et al. 2018b S1	OAP1	0.17	405	0.05	Positive Emotions	Positive Affect	Approach	OAP
Méndez-Giménez et al. 2018b S1	OAV1	0.26	405	0.05	Positive Emotions	Positive Affect	Avoidance	OAV
Méndez-Giménez et al. 2018b S1	SAP1	0.32	405	0.04	Positive Emotions	Positive Affect	Approach	SAP
Méndez-Giménez et al. 2018b S1	SAV1	0.26	405	0.05	Positive Emotions	Positive Affect	Avoidance	SAV
Méndez-Giménez et al. 2018b S1	TAP1	0.32	405	0.04	Positive Emotions	Positive Affect	Approach	TAP
Méndez-Giménez et al. 2018b S1	TAV1	0.30	405	0.05	Positive Emotions	Positive Affect	Avoidance	TAV
Méndez-Giménez et al. 2018b S2	OAP4	0.12	646	0.04	Negative Emotions	Negative Affect	Approach	OAP
Méndez-Giménez et al. 2018b S2	OAV4	0.06	646	0.04	Negative Emotions	Negative Affect	Avoidance	OAV
Méndez-Giménez et al. 2018b S2	SAP4	-0.08	646	0.04	Negative Emotions	Negative Affect	Approach	SAP
Méndez-Giménez et al. 2018b S2	SAV4	-0.03	646	0.04	Negative Emotions	Negative Affect	Avoidance	SAV
Méndez-Giménez et al. 2018b S2	TAP4	0.10	646	0.04	Negative Emotions	Negative Affect	Approach	TAP
Méndez-Giménez et al. 2018b S2	TAV4	0.04	646	0.04	Negative Emotions	Negative Affect	Avoidance	TAV
Méndez-Giménez et al. 2018b S2	OAP3	0.21	646	0.04	Positive Emotions	Positive Affect	Approach	OAP
Méndez-Giménez et al. 2018b S2	OAV3	0.18	646	0.04	Positive Emotions	Positive Affect	Avoidance	OAV
Méndez-Giménez et al. 2018b S2	SAP3	0.37	646	0.03	Positive Emotions	Positive Affect	Approach	SAP

Méndez-Giménez et al. 2018b S2	SAV3	0.27	646	0.04	Positive Emotions	Positive Affect	Avoidance	SAV
Méndez-Giménez et al. 2018b S2	TAP3	0.26	646	0.04	Positive Emotions	Positive Affect	Approach	TAP
Méndez-Giménez et al. 2018b S2	TAV3	0.27	646	0.04	Positive Emotions	Positive Affect	Avoidance	TAV
Méndez-Giménez et al. 2018b S3	OAP6	0.00	559	0.04	Negative Emotions	Negative Affect	Approach	OAP
Méndez-Giménez et al. 2018b S3	OAV6	0.00	559	0.04	Negative Emotions	Negative Affect	Avoidance	OAV
Méndez-Giménez et al. 2018b S3	SAP6	-0.06	559	0.04	Negative Emotions	Negative Affect	Approach	SAP
Méndez-Giménez et al. 2018b S3	SAV6	-0.03	559	0.04	Negative Emotions	Negative Affect	Avoidance	SAV
Méndez-Giménez et al. 2018b S3	TAP6	0.00	559	0.04	Negative Emotions	Negative Affect	Approach	TAP
Méndez-Giménez et al. 2018b S3	TAV6	0.08	559	0.04	Negative Emotions	Negative Affect	Avoidance	TAV
Méndez-Giménez et al. 2018b S3	OAP5	0.19	559	0.04	Positive Emotions	Positive Affect	Approach	OAP
Méndez-Giménez et al. 2018b S3	OAV5	0.23	559	0.04	Positive Emotions	Positive Affect	Avoidance	OAV
Méndez-Giménez et al. 2018b S3	SAP5	0.35	559	0.04	Positive Emotions	Positive Affect	Approach	SAP
Méndez-Giménez et al. 2018b S3	SAV5	0.31	559	0.04	Positive Emotions	Positive Affect	Avoidance	SAV
Méndez-Giménez et al. 2018b S3	TAP5	0.31	559	0.04	Positive Emotions	Positive Affect	Approach	TAP
Méndez-Giménez et al. 2018b S3	TAV5	0.29	559	0.04	Positive Emotions	Positive Affect	Avoidance	TAV
Méndez-Giménez et al. 2018c	OAP1M	0.06	2284	0.02	Motivation	Autonomy Index	Approach	OAP
Méndez-Giménez et al. 2018c	OAV1M	0.11	2284	0.02	Motivation	Autonomy Index	Avoidance	OAV
Méndez-Giménez et al. 2018c	SAP1M	0.53	2284	0.02	Motivation	Autonomy Index	Approach	SAP
Méndez-Giménez et al. 2018c	SAV1M	0.31	2284	0.02	Motivation	Autonomy Index	Avoidance	SAV
Méndez-Giménez et al. 2018c	TAP1M	0.35	2284	0.02	Motivation	Autonomy Index	Approach	TAP
Méndez-Giménez et al. 2018c	TAV1M	0.24	2284	0.02	Motivation	Autonomy Index	Avoidance	TAV

Rivera-Pérez et al. 2021 B	SAP1L	0.35	1328	0.02	Learning	Group Processing	Approach	SAP
Rivera-Pérez et al. 2021 B	SAP2L	0.40	1328	0.02	Learning	Positive Interdependence	Approach	SAP
Rivera-Pérez et al. 2021 B	SAP3L	0.37	1328	0.02	Learning	Promotive Interaction	Approach	SAP
Rivera-Pérez et al. 2021 B	SAP4L	0.46	1328	0.02	Learning	Individual Accountability	Approach	SAP
Rivera-Pérez et al. 2021 B	SAP5L	0.32	1328	0.02	Learning	Interpersonal Skills	Approach	SAP
Rivera-Pérez et al. 2021 B	TAP1L	0.30	1328	0.02	Learning	Group Processing	Approach	TAP
Rivera-Pérez et al. 2021 B	TAP2L	0.39	1328	0.02	Learning	Positive Interdependence	Approach	TAP
Rivera-Pérez et al. 2021 B	TAP3L	0.35	1328	0.02	Learning	Promotive Interaction	Approach	TAP
Rivera-Pérez et al. 2021 B	TAP4L	0.48	1328	0.02	Learning	Individual Accountability	Approach	TAP
Rivera-Pérez et al. 2021 B	TAP5L	0.29	1328	0.03	Learning	Interpersonal Skills	Approach	TAP
Sari et al. 2020	OAP1L	0.30	424	0.04	Learning	Effort	Approach	OAP
Sari et al. 2020	OAP2L	0.33	424	0.04	Learning	Concentration	Approach	OAP
Sari et al. 2020	OAV1L	-0.12	424	0.05	Learning	Effort	Avoidance	OAV
Sari et al. 2020	OAV2L	-0.18	424	0.05	Learning	Concentration	Avoidance	OAV
Sari et al. 2020	TAP1L	0.68	424	0.03	Learning	Effort	Approach	TAP
Sari et al. 2020	TAP2L	0.60	424	0.03	Learning	Concentration	Approach	TAP
Sari et al. 2020	TAV1L	0.60	424	0.03	Learning	Effort	Avoidance	TAV
Sari et al. 2020	TAV2L	0.47	424	0.04	Learning	Concentration	Avoidance	TAV
Sari et al. 2020	OAP2	-0.08	424	0.05	Negative Emotions	Anxiety	Approach	OAP
Sari et al. 2020	OAV2	0.16	424	0.05	Negative Emotions	Anxiety	Avoidance	OAV
Sari et al. 2020	TAP2	-0.20	424	0.05	Negative Emotions	Anxiety	Approach	TAP
Sari et al. 2020	TAV2	-0.18	424	0.05	Negative Emotions	Anxiety	Avoidance	TAV
Sari et al. 2020	OAP1	0.33	424	0.04	Positive Emotions	Enjoyment	Approach	OAP
Sari et al. 2020	OAV1	-0.24	424	0.05	Positive Emotions	Enjoyment	Avoidance	OAV
Sari et al. 2020	TAP1	0.60	424	0.03	Positive Emotions	Enjoyment	Approach	TAP
Sari et al. 2020	TAV1	0.70	424	0.02	Positive Emotions	Enjoyment	Avoidance	TAV
Shen et al. 2020	OAP1IP	-0.13	792	0.03	Performance	50m Dash	Approach	OAP

Shen et al. 2020	OAP2P	0.13	792	0.03	Performance	Standing Long Jump	Approach	OAP
Shen et al. 2020	OAV1IP	0.04	792	0.04	Performance	50m Dash	Avoidance	OAV
Shen et al. 2020	OAV2P	0.03	792	0.04	Performance	Standing Long Jump	Avoidance	OAV
Shen et al. 2020	SAP1IP	-0.12	792	0.04	Performance	50m Dash	Approach	SAP
Shen et al. 2020	SAP2P	0.14	792	0.03	Performance	Standing Long Jump	Approach	SAP
Shen et al. 2020	SAV1IP	0.01	792	0.04	Performance	50m Dash	Avoidance	SAV
Shen et al. 2020	SAV2P	0.02	792	0.04	Performance	Standing Long Jump	Avoidance	SAV
Shen et al. 2020	TAP1IP	-0.10	792	0.04	Performance	50m Dash	Approach	TAP
Shen et al. 2020	TAP2P	0.14	792	0.03	Performance	Standing Long Jump	Approach	TAP
Shen et al. 2020	TAV1IP	0.01	792	0.04	Performance	50m Dash	Avoidance	TAV
Shen et al. 2020	TAV2P	0.01	792	0.04	Performance	Standing Long Jump	Avoidance	TAV
Thomas 2022	OAP2L	0.21	482	0.04	Learning	Task-Value	Approach	OAP
Thomas 2022	OAP3L	0.27	482	0.04	Learning	Self-Efficacy	Approach	OAP
Thomas 2022	OAV2L	0.25	482	0.04	Learning	Task-Value	Avoidance	OAV
Thomas 2022	OAV3L	0.28	482	0.04	Learning	Self-Efficacy	Avoidance	OAV
Thomas 2022	SAP2L	0.37	482	0.04	Learning	Task-Value	Approach	SAP
Thomas 2022	SAP3L	0.34	482	0.04	Learning	Self-Efficacy	Approach	SAP
Thomas 2022	SAV2L	0.26	482	0.04	Learning	Task-Value	Avoidance	SAV
Thomas 2022	SAV3L	0.23	482	0.04	Learning	Self-Efficacy	Avoidance	SAV
Thomas 2022	TAP2L	0.39	482	0.04	Learning	Task-Value	Approach	TAP
Thomas 2022	TAP3L	0.42	482	0.04	Learning	Self-Efficacy	Approach	TAP
Thomas 2022	TAV2L	0.35	482	0.04	Learning	Task-Value	Avoidance	TAV
Thomas 2022	TAV3L	0.36	482	0.04	Learning	Self-Efficacy	Avoidance	TAV
Thomas 2022	OAP1L	0.08	482	0.05	Learning (D)	Test Irrelevant Thinking	Approach	OAP
Thomas 2022	OAV1L	0.10	482	0.05	Learning (D)	Test Irrelevant Thinking	Avoidance	OAV

Thomas 2022	SAP1L	0.00	482	0.05	Learning (D)	Test Irrelevant Thinking	Approach	SAP
Thomas 2022	SAV1L	0.00	482	0.05	Learning (D)	Test Irrelevant Thinking	Avoidance	SAV
Thomas 2022	TAP1L	-0.14	482	0.04	Learning (D)	Test Irrelevant Thinking	Approach	TAP
Thomas 2022	TAV1L	-0.06	482	0.05	Learning (D)	Test Irrelevant Thinking	Avoidance	TAV
Thomas 2022	OAP1	0.11	482	0.05	Negative Emotions	Worry	Approach	OAP
Thomas 2022	OAP2	0.10	482	0.05	Negative Emotions	Tension	Approach	OAP
Thomas 2022	OAP3	0.09	482	0.05	Negative Emotions	Bodily Symptoms	Approach	OAP
Thomas 2022	OAV1	0.14	482	0.04	Negative Emotions	Worry	Avoidance	OAV
Thomas 2022	OAV2	0.11	482	0.05	Negative Emotions	Tension	Avoidance	OAV
Thomas 2022	OAV3	0.08	482	0.05	Negative Emotions	Bodily Symptoms	Avoidance	OAV
Thomas 2022	SAP1	0.08	482	0.05	Negative Emotions	Worry	Approach	SAP
Thomas 2022	SAP2	0.11	482	0.05	Negative Emotions	Tension	Approach	SAP
Thomas 2022	SAP3	0.03	482	0.05	Negative Emotions	Bodily Symptoms	Approach	SAP
Thomas 2022	SAV1	0.09	482	0.05	Negative Emotions	Worry	Avoidance	SAV
Thomas 2022	SAV2	0.11	482	0.05	Negative Emotions	Tension	Avoidance	SAV
Thomas 2022	SAV3	0.03	482	0.05	Negative Emotions	Bodily Symptoms	Avoidance	SAV
Thomas 2022	TAP1	-0.06	482	0.05	Negative Emotions	Worry	Approach	TAP
Thomas 2022	TAP2	0.01	482	0.05	Negative Emotions	Tension	Approach	TAP
Thomas 2022	TAP3	-0.07	482	0.05	Negative Emotions	Bodily Symptoms	Approach	TAP
Thomas 2022	TAV1	0.01	482	0.05	Negative Emotions	Worry	Avoidance	TAV
Thomas 2022	TAV2	0.07	482	0.05	Negative Emotions	Tension	Avoidance	TAV
Thomas 2022	TAV3	-0.03	482	0.05	Negative Emotions	Bodily Symptoms	Avoidance	TAV
Üztemur 2020	OAP1L	-0.29	259	0.06	Learning	Deep Learning	Approach	OAP
Üztemur 2020	OAV1L	-0.20	259	0.06	Learning	Deep Learning	Avoidance	OAV
Üztemur 2020	SAP1L	0.69	259	0.03	Learning	Deep Learning	Approach	SAP
Üztemur 2020	SAV1L	0.12	259	0.06	Learning	Deep Learning	Avoidance	SAV
Üztemur 2020	TAP1L	0.38	259	0.05	Learning	Deep Learning	Approach	TAP
Üztemur 2020	TAV1L	0.47	259	0.05	Learning	Deep Learning	Avoidance	TAV

Üztemur 2020	OAP2L	0.41	259	0.05	Learning (D)	Surface Learning	Approach	OAP
Üztemur 2020	OAV2L	0.32	259	0.06	Learning (D)	Surface Learning	Avoidance	OAV
Üztemur 2020	SAP2L	-0.55	259	0.04	Learning (D)	Surface Learning	Approach	SAP
Üztemur 2020	SAV2L	0.00	259	0.06	Learning (D)	Surface Learning	Avoidance	SAV
Üztemur 2020	TAP2L	-0.29	259	0.06	Learning (D)	Surface Learning	Approach	TAP
Üztemur 2020	TAV2L	-0.33	259	0.06	Learning (D)	Surface Learning	Avoidance	TAV
Van Yperen 2022	OAP1M	0.27	647	0.04	Motivation	Competence Satisfaction	Approach	OAP
Van Yperen 2022	OAV1M	0.09	647	0.04	Motivation	Competence Satisfaction	Avoidance	OAV
Van Yperen 2022	SAP1M	0.26	647	0.04	Motivation	Competence Satisfaction	Approach	SAP
Van Yperen 2022	SAV1M	0.06	647	0.04	Motivation	Competence Satisfaction	Avoidance	SAV
Van Yperen 2022	TAP1M	0.23	647	0.04	Motivation	Competence Satisfaction	Approach	TAP
Van Yperen 2022	TAV1M	0.06	647	0.04	Motivation	Competence Satisfaction	Avoidance	TAV
Van Yperen 2022	OAP1	0.21	647	0.04	Positive Emotions	Satisfaction with Win	Approach	OAP
Van Yperen 2022	OAP2	-0.28	647	0.04	Positive Emotions	Satisfaction with Best Performance	Approach	OAP
Van Yperen 2022	OAV1	0.18	647	0.04	Positive Emotions	Satisfaction with Win	Avoidance	OAV
Van Yperen 2022	OAV2	-0.14	647	0.04	Positive Emotions	Satisfaction with Best Performance	Avoidance	OAV
Van Yperen 2022	SAP1	0.06	647	0.04	Positive Emotions	Satisfaction with Win	Approach	SAP
Van Yperen 2022	SAP2	-0.05	647	0.04	Positive Emotions	Satisfaction with Best Performance	Approach	SAP
Van Yperen 2022	SAV1	-0.04	647	0.04	Positive Emotions	Satisfaction with Win	Avoidance	SAV
Van Yperen 2022	SAV2	0.02	647	0.04	Positive Emotions	Satisfaction with Best Performance	Avoidance	SAV
Van Yperen 2022	TAP1	0.03	647	0.04	Positive Emotions	Satisfaction with Win	Approach	TAP

Van Yperen 2022	TAP2	-0.06	647	0.04	Positive Emotions	Satisfaction with Best Performance	Approach	TAP
Van Yperen 2022	TAV1	-0.03	647	0.04	Positive Emotions	Satisfaction with Win	Avoidance	TAV
Van Yperen 2022	TAV2	-0.04	647	0.04	Positive Emotions	Satisfaction with Best Performance	Avoidance	TAV
Wang et al. 2017	OAP12D	0.11	475	0.05	Individual Difference	Incremental Theory	Approach	OAP
Wang et al. 2017	OAP1ID	0.08	475	0.05	Individual Difference	Entity Theory	Approach	OAP
Wang et al. 2017	OAV12D	-0.07	475	0.05	Individual Difference	Incremental Theory	Avoidance	OAV
Wang et al. 2017	OAV1ID	0.23	475	0.04	Individual Difference	Entity Theory	Avoidance	OAV
Wang et al. 2017	SAP12D	0.46	475	0.04	Individual Difference	Incremental Theory	Approach	SAP
Wang et al. 2017	SAP1ID	-0.24	475	0.04	Individual Difference	Entity Theory	Approach	SAP
Wang et al. 2017	SAV12D	0.16	475	0.04	Individual Difference	Incremental Theory	Avoidance	SAV
Wang et al. 2017	SAV1ID	0.02	475	0.05	Individual Difference	Entity Theory	Avoidance	SAV
Wang et al. 2017	TAP12D	0.54	475	0.03	Individual Difference	Incremental Theory	Approach	TAP
Wang et al. 2017	TAP1ID	-0.29	475	0.04	Individual Difference	Entity Theory	Approach	TAP
Wang et al. 2017	TAV12D	0.30	475	0.04	Individual Difference	Incremental Theory	Avoidance	TAV
Wang et al. 2017	TAV1ID	-0.17	475	0.04	Individual Difference	Entity Theory	Avoidance	TAV
Wang et al. 2017	OAP1M	0.08	475	0.05	Motivation	Intrinsic Motivation	Approach	OAP
Wang et al. 2017	OAP2M	0.15	475	0.04	Motivation	Perceived Competence	Approach	OAP
Wang et al. 2017	OAV1M	-0.07	475	0.05	Motivation	Intrinsic Motivation	Avoidance	OAV
Wang et al. 2017	OAV2M	-0.07	475	0.05	Motivation	Perceived Competence	Avoidance	OAV
Wang et al. 2017	SAP1M	0.40	475	0.04	Motivation	Intrinsic Motivation	Approach	SAP

Wang et al. 2017	SAP2M	0.24	475	0.04	Motivation	Perceived Competence	Approach	SAP
Wang et al. 2017	SAV1M	0.19	475	0.04	Motivation	Intrinsic Motivation	Avoidance	SAV
Wang et al. 2017	SAV2M	-0.05	475	0.05	Motivation	Perceived Competence	Avoidance	SAV
Wang et al. 2017	TAP1M	0.49	475	0.03	Motivation	Intrinsic Motivation	Approach	TAP
Wang et al. 2017	TAP2M	0.29	475	0.04	Motivation	Perceived Competence	Approach	TAP
Wang et al. 2017	TAV1M	0.29	475	0.04	Motivation	Intrinsic Motivation	Avoidance	TAV
Wang et al. 2017	TAV2M	0.20	475	0.04	Motivation	Perceived Competence	Avoidance	TAV
Wei et al. 2020	OAP1	0.26	406	0.05	Positive Emotions	Psychological Well-Being	Approach	OAP
Wei et al. 2020	OAV1	0.23	406	0.05	Positive Emotions	Psychological Well-Being	Avoidance	OAV
Wei et al. 2020	SAP1	0.37	406	0.04	Positive Emotions	Psychological Well-Being	Approach	SAP
Wei et al. 2020	SAV1	0.28	406	0.05	Positive Emotions	Psychological Well-Being	Avoidance	SAV
Wei et al. 2020	TAP1	0.43	406	0.04	Positive Emotions	Psychological Well-Being	Approach	TAP
Wei et al. 2020	TAV1	0.14	406	0.05	Positive Emotions	Psychological Well-Being	Avoidance	TAV
Yang & Cao 2013	OAP1M	0.18	93	0.10	Motivation	Intrinsic Motivation	Approach	OAP
Yang & Cao 2013	SAP1M	0.40	93	0.09	Motivation	Intrinsic Motivation	Approach	SAP
Yang & Cao 2013	TAP1M	0.30	93	0.10	Motivation	Intrinsic Motivation	Approach	TAP
Yang & Cao 2013	OAP2M	0.50	93	0.08	Motivation (Ext)	Extrinsic Motivation	Approach	OAP
Yang & Cao 2013	SAP2M	0.33	93	0.09	Motivation (Ext)	Extrinsic Motivation	Approach	SAP
Yang & Cao 2013	TAP2M	0.24	93	0.10	Motivation (Ext)	Extrinsic Motivation	Approach	TAP
Zhou et al. 2022 S2	OAP1P	0.42	348	0.04	Performance	Academic	Approach	OAP
Zhou et al. 2022 S2	OAV1P	-0.15	348	0.05	Performance	Academic	Avoidance	OAV
Zhou et al. 2022 S2	SAP1P	0.38	348	0.05	Performance	Academic	Approach	SAP

Zhou et al. 2022 S2	SAV1P	-0.13	348	0.05	Performance	Academic	Avoidance	SAV
Zhou et al. 2022 S2	TAP1P	0.35	348	0.05	Performance	Academic	Approach	TAP
Zhou et al. 2022 S2	TAV1P	0.10	348	0.05	Performance	Academic	Avoidance	TAV

Supplement file. All correlate random-effects funnel plots.

- Approach and Avoidance Achievement Goals and Correlates.

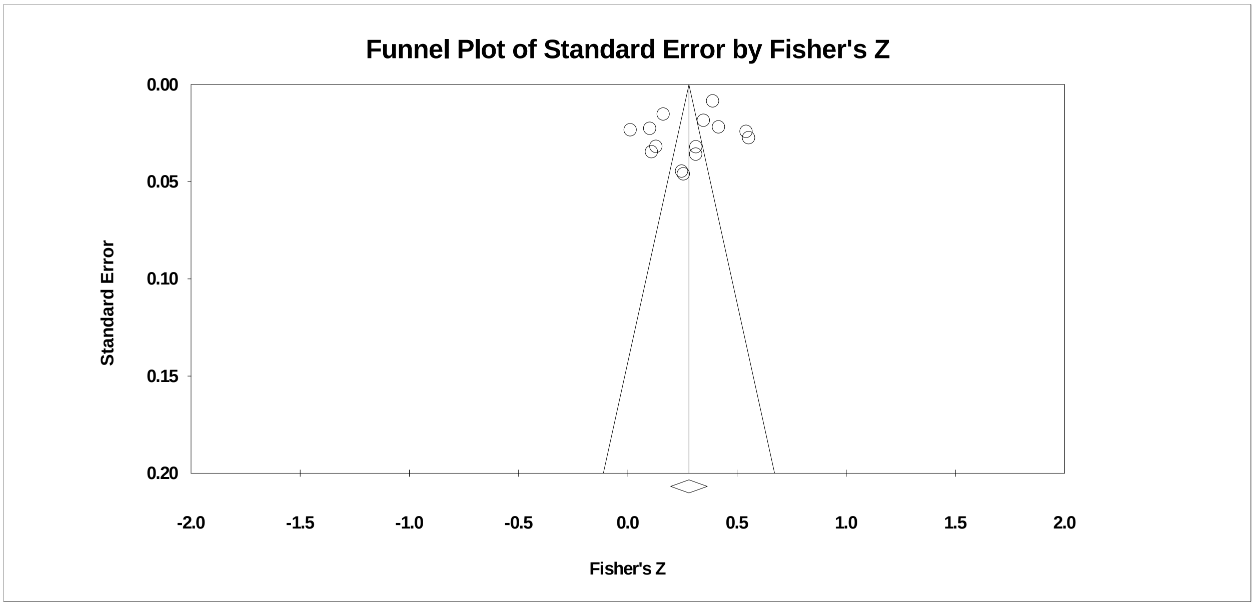

Figure S1. Approach goals and facilitative learning strategies random-effects funnel plot.

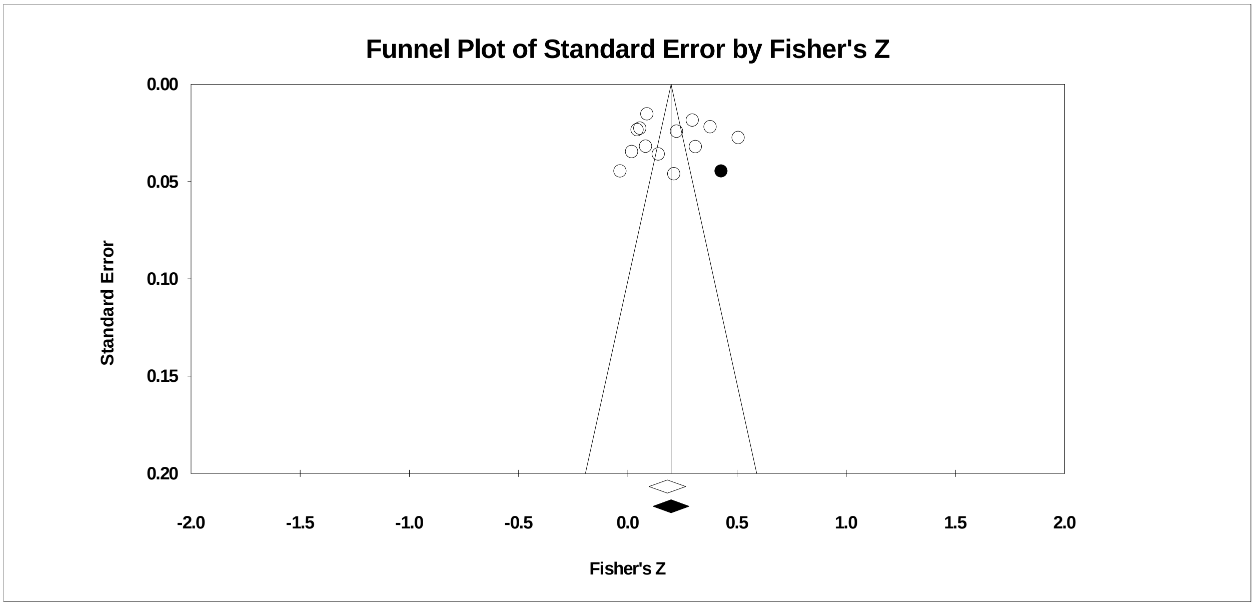

Figure S2. Avoidance goals and facilitative learning strategies random-effects funnel plot.

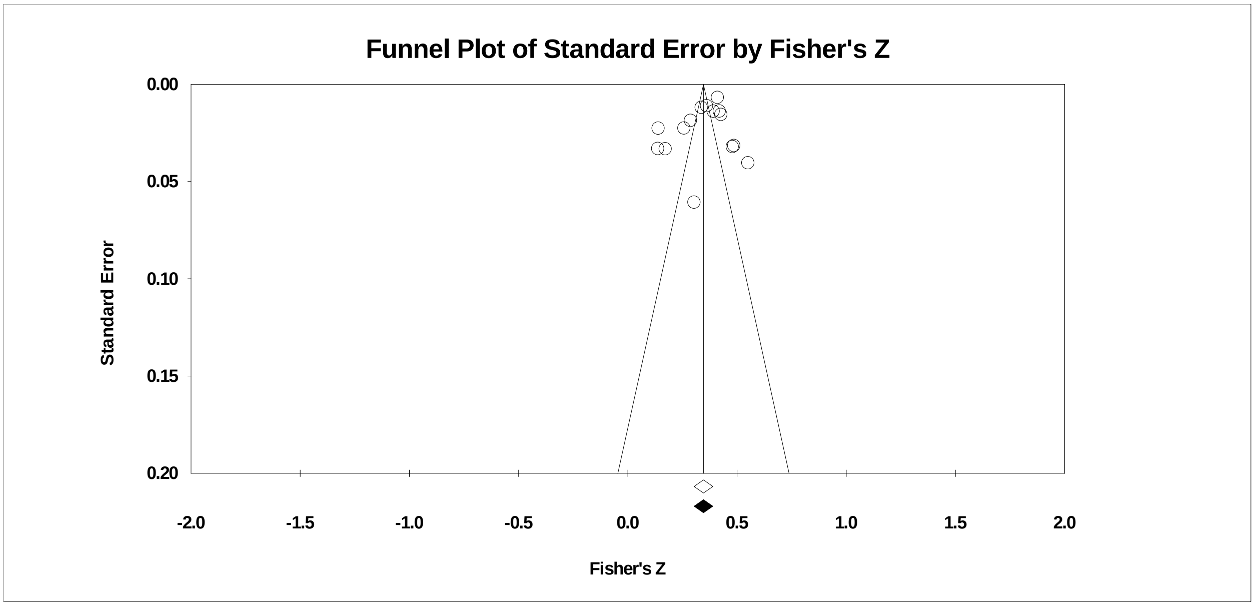

Figure S3. Approach goals and desired motivations random-effects funnel plot.

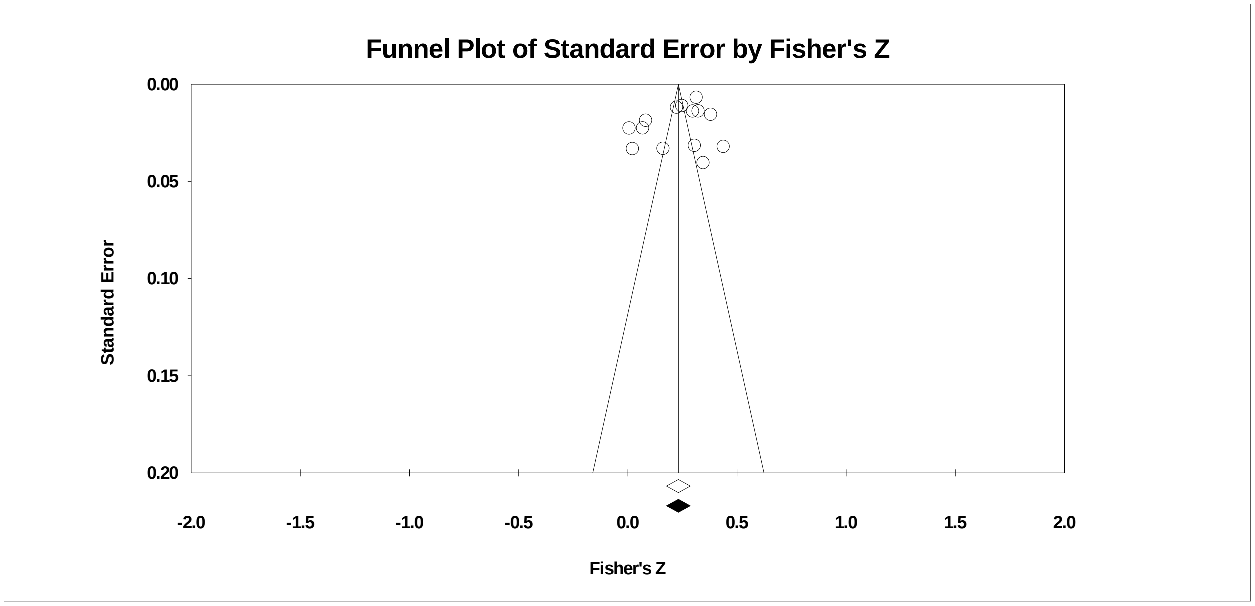

Figure S4. Avoidance goals and desired motivations random-effects funnel plot.

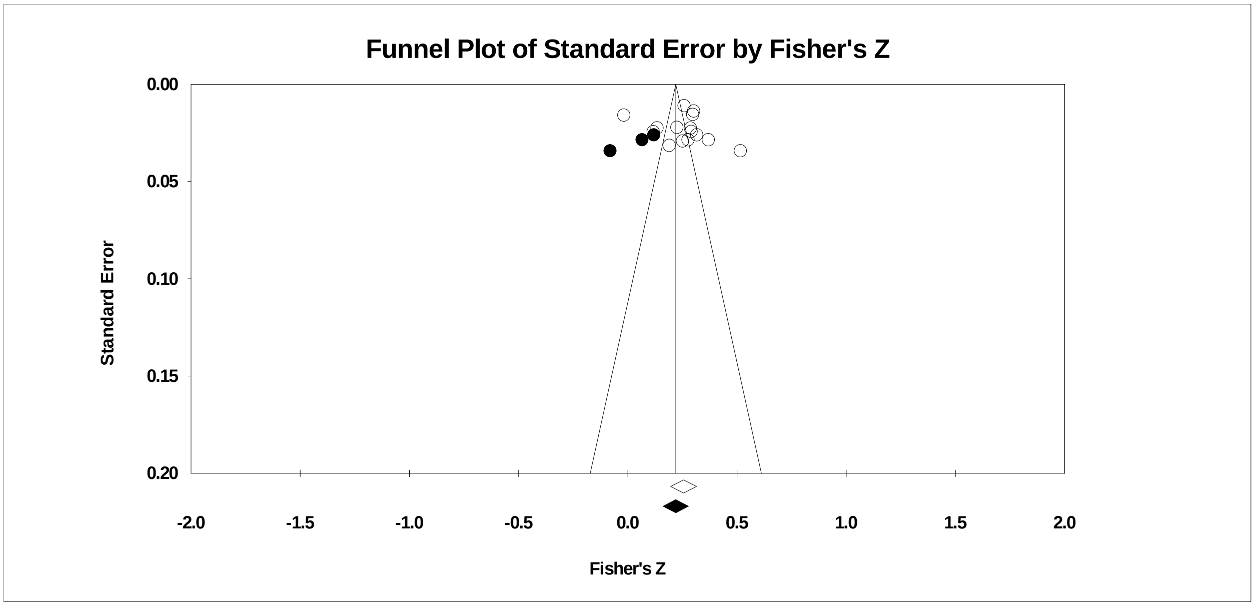

Figure S5. Approach goals and positive emotions random-effects funnel plot.

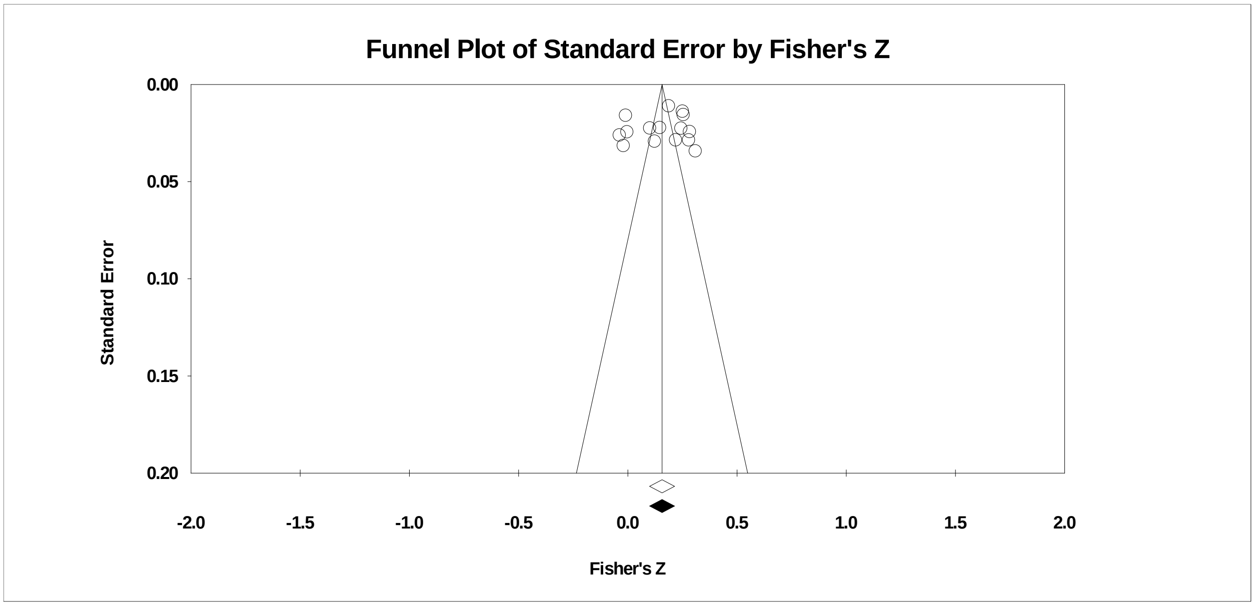

Figure S6. Avoidance goals and positive emotions random-effects funnel plot.

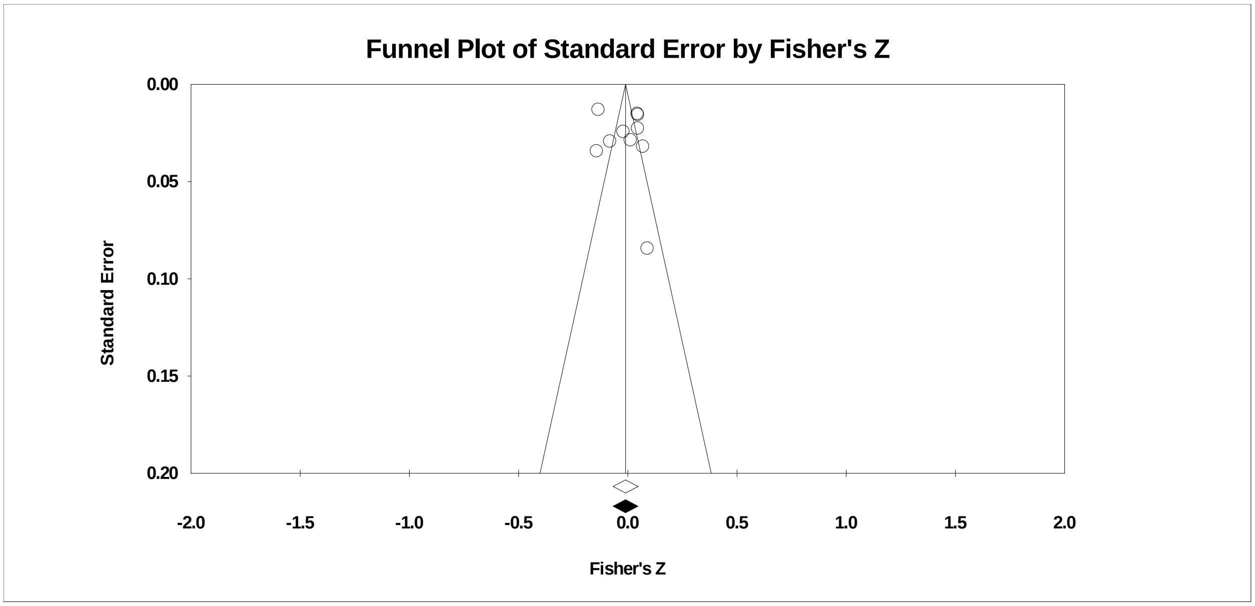

Figure S7. Approach goals and negative emotions random-effects funnel plot.

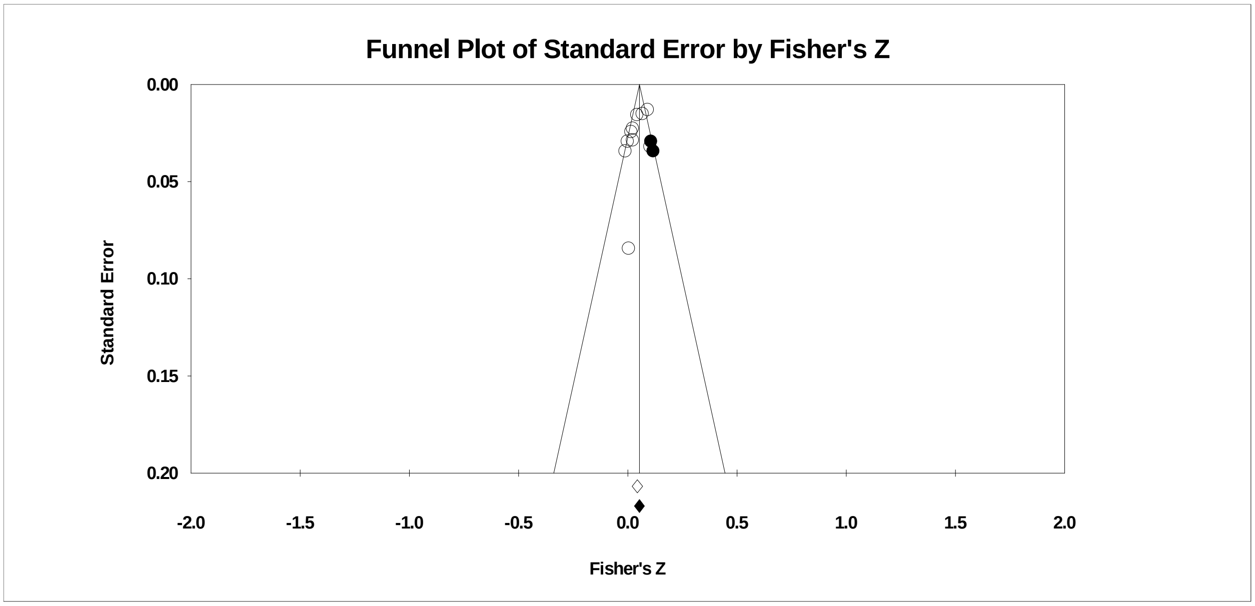

Figure S8. Avoidance goals and negative emotions random-effects funnel plot.

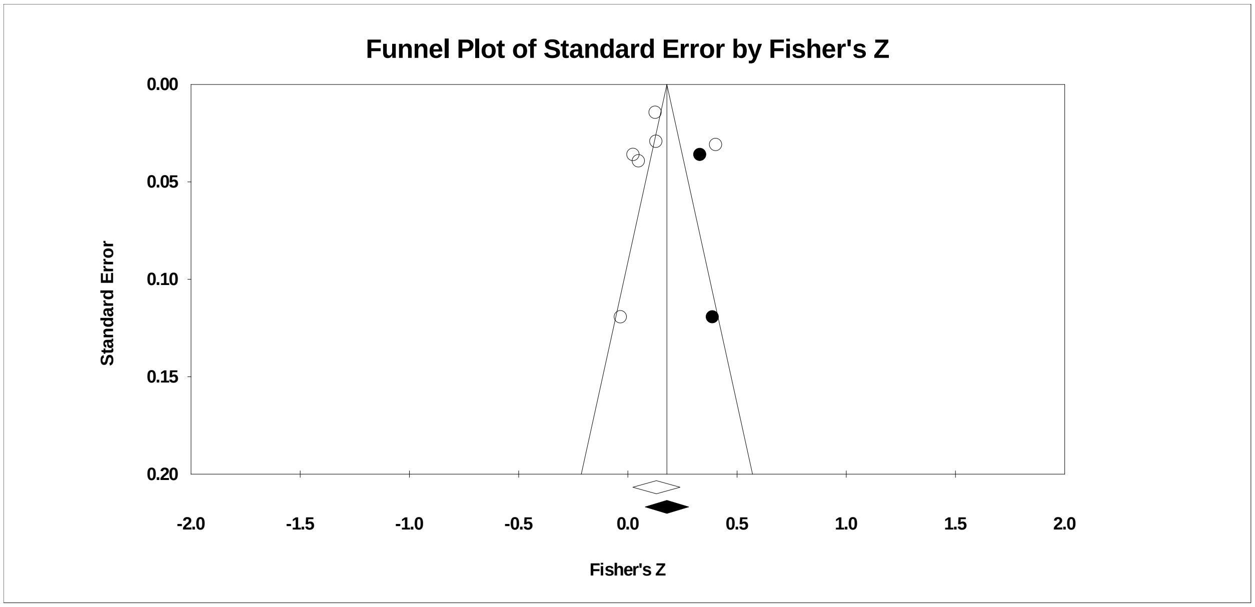

Figure S9. Approach goals and performance random-effects funnel plot.

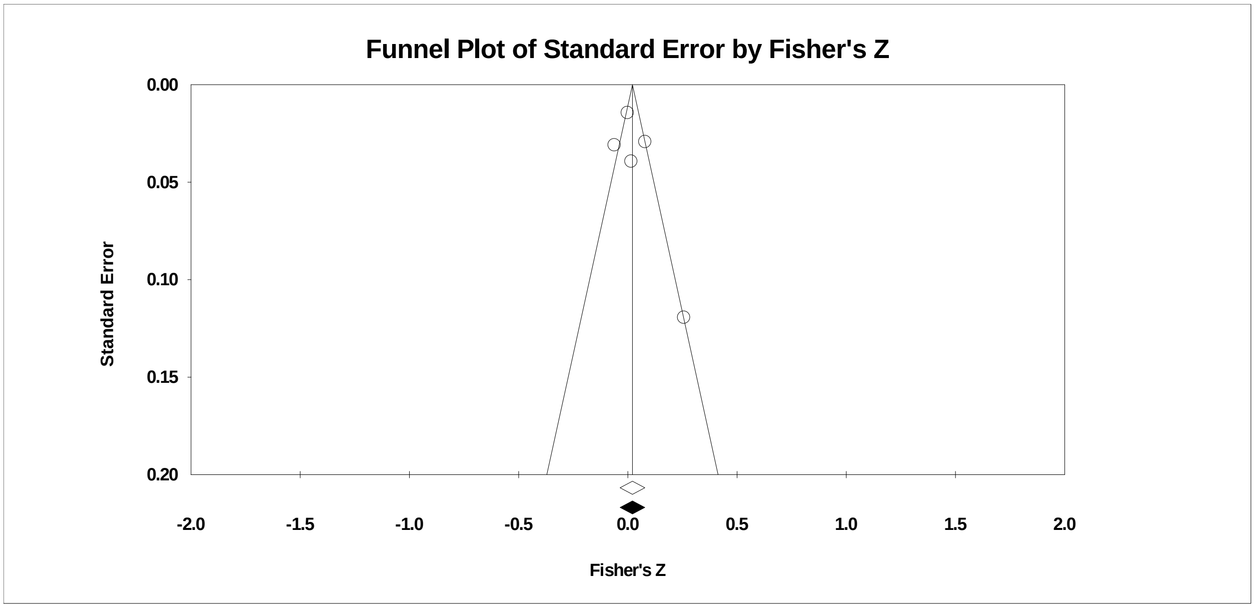

Figure S10. Avoidance goals and performance random-effects funnel plot.

- Task achievement goals and correlates.

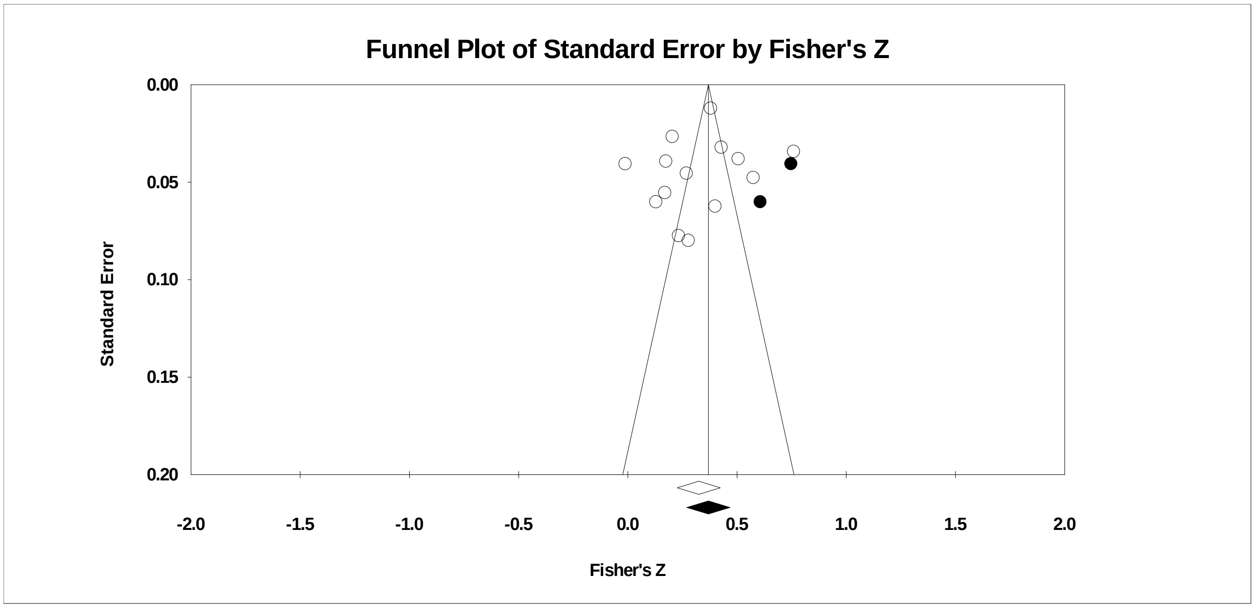

Figure S11. Task approach goal and facilitative learning strategies random-effects funnel plot.

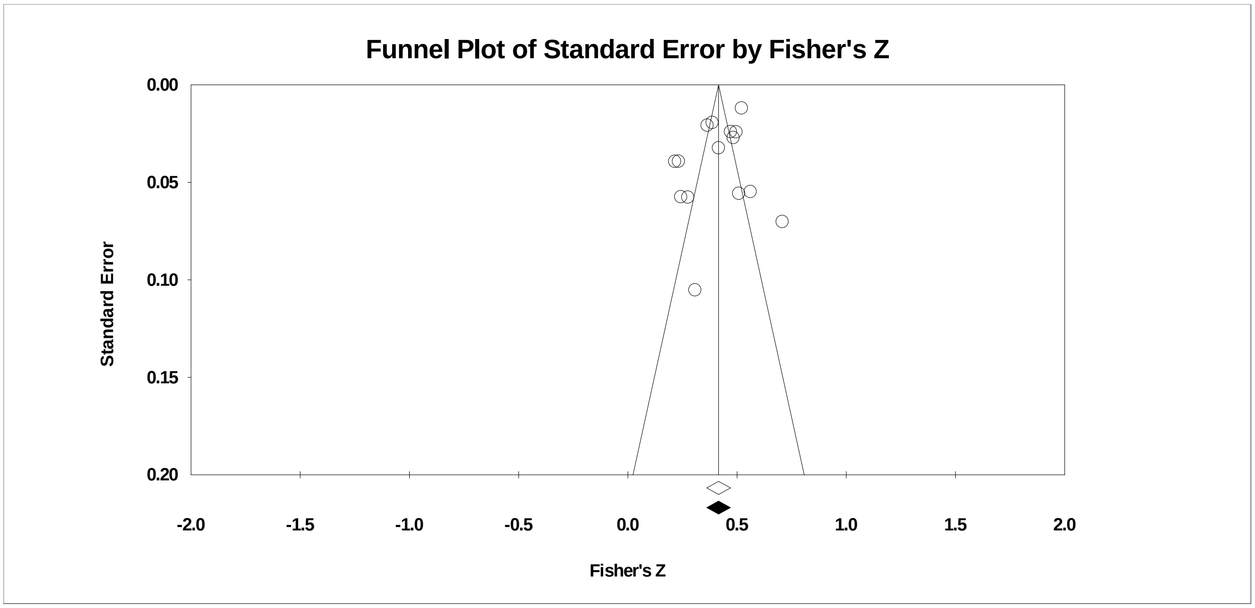

Figure S12. Task approach goal and desired motivations random-effects funnel plot.

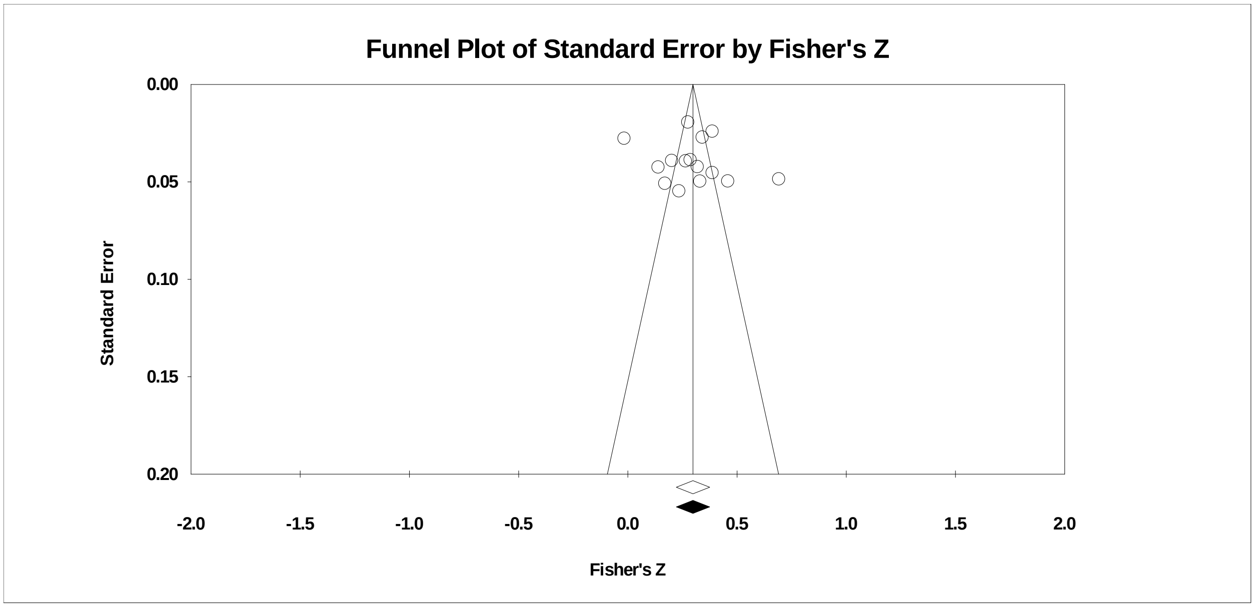

Figure S13. Task approach goal and positive emotions strategies random-effects funnel plot.

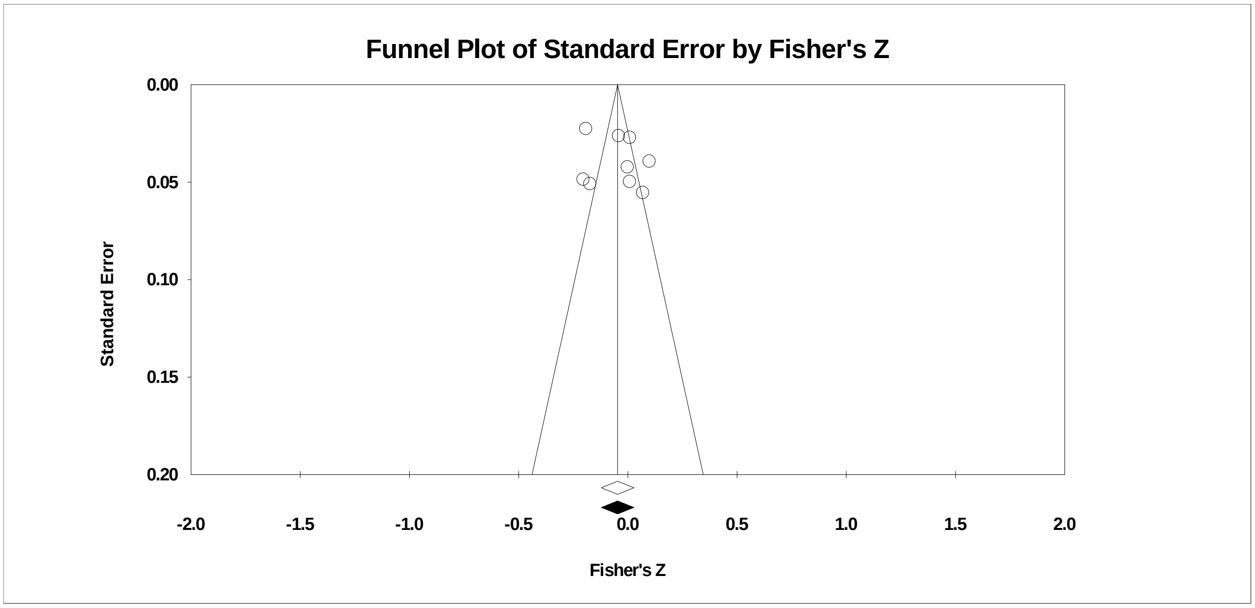

Figure S14. Task approach goal and negative emotions random-effects funnel plot.

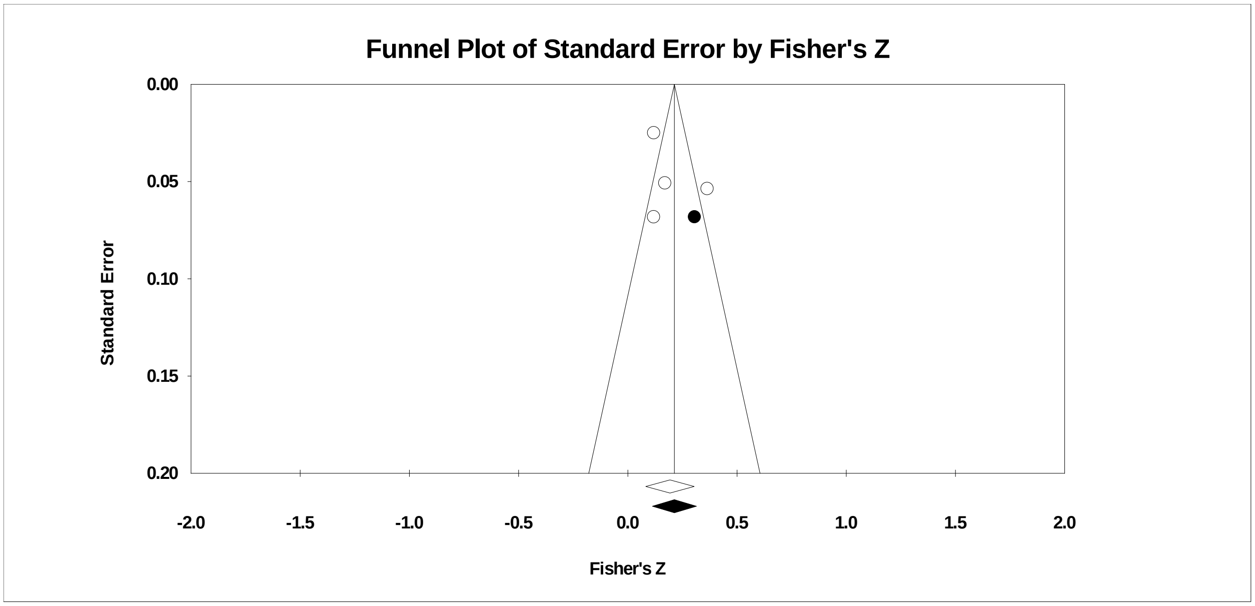

Figure S15. Task approach goal and performance random-effects funnel plot.

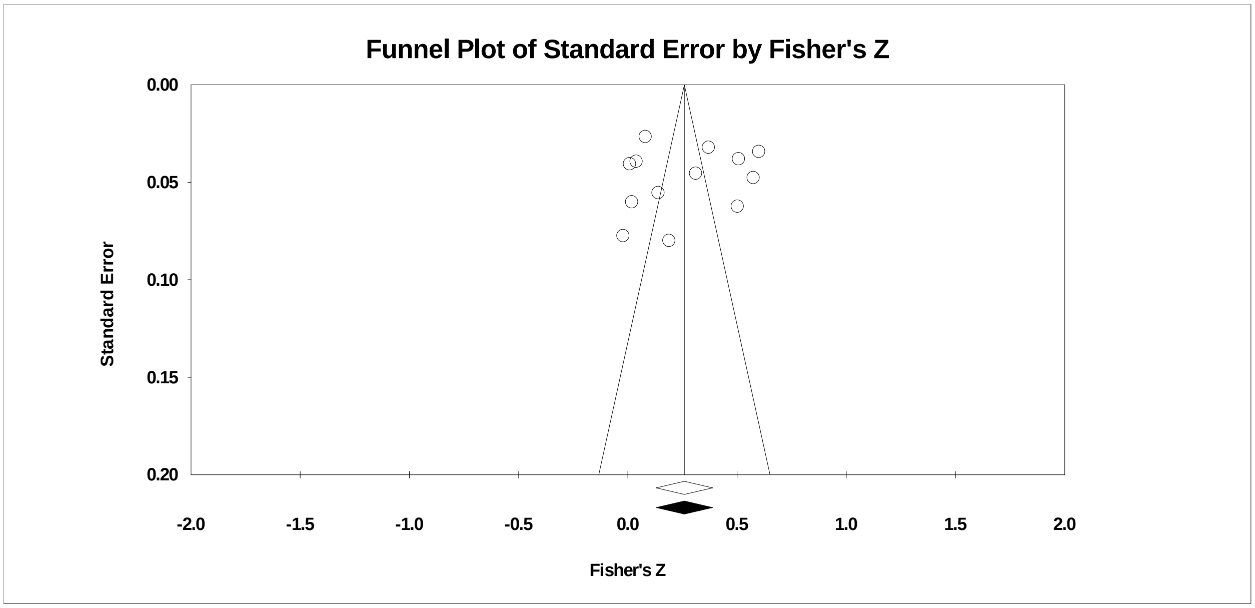

Figure S16. Task avoidance goal and facilitative learning strategies random-effects funnel plot.

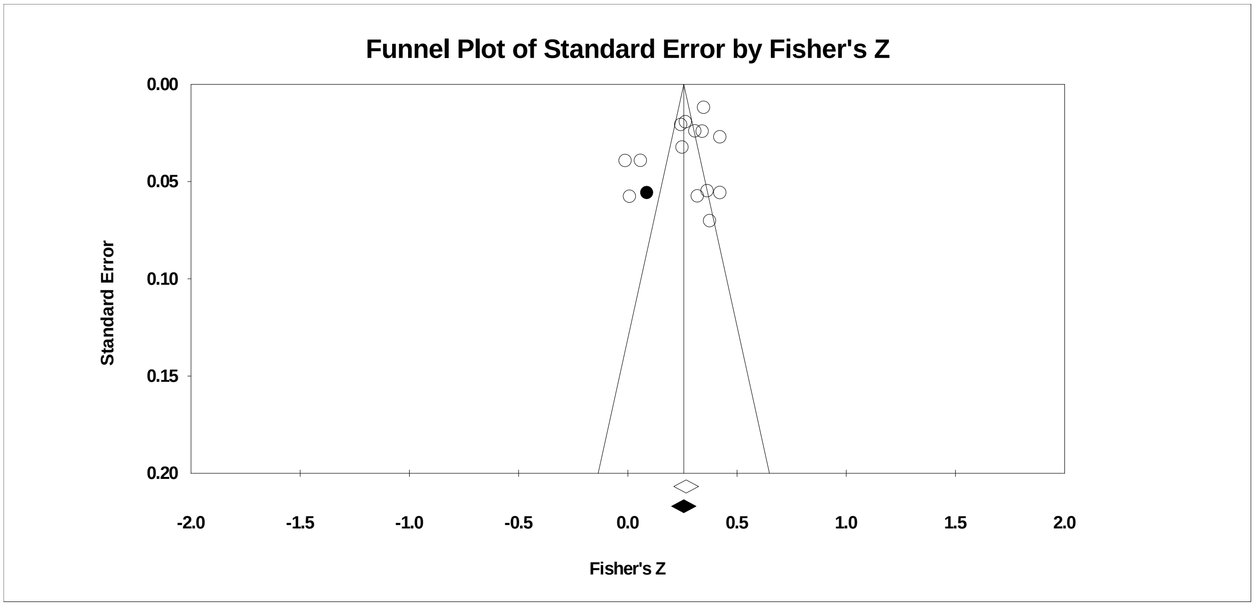

Figure S17. Task avoidance goal and desired motivations random-effects funnel plot.

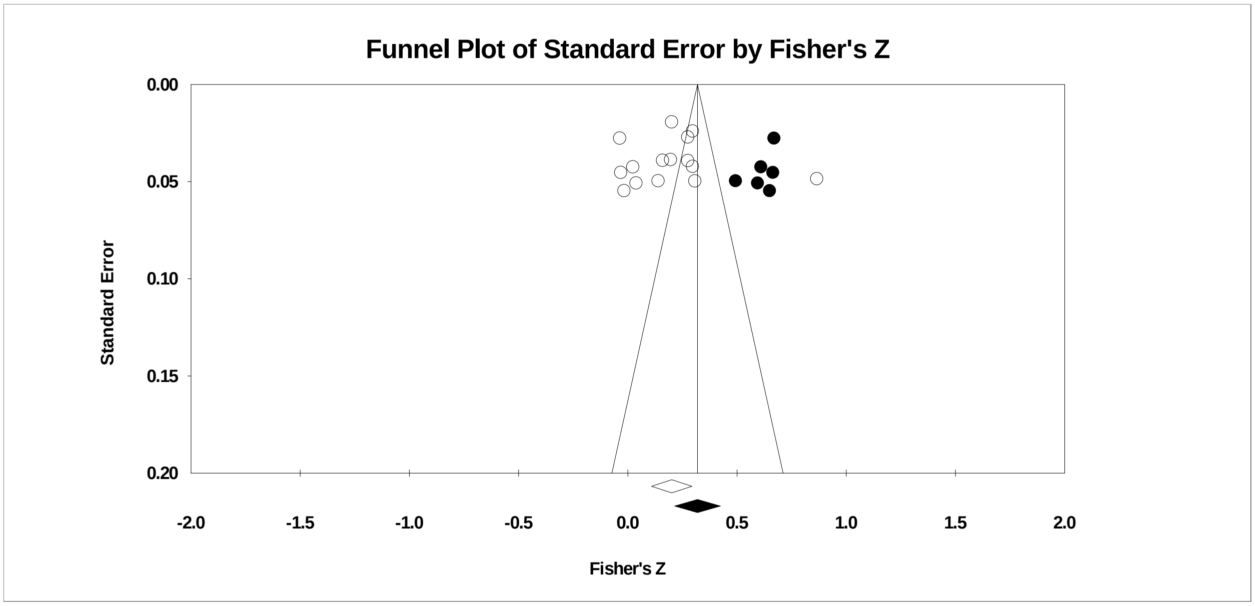

Figure S18. Task avoidance goal and positive emotions strategies random-effects funnel plot.

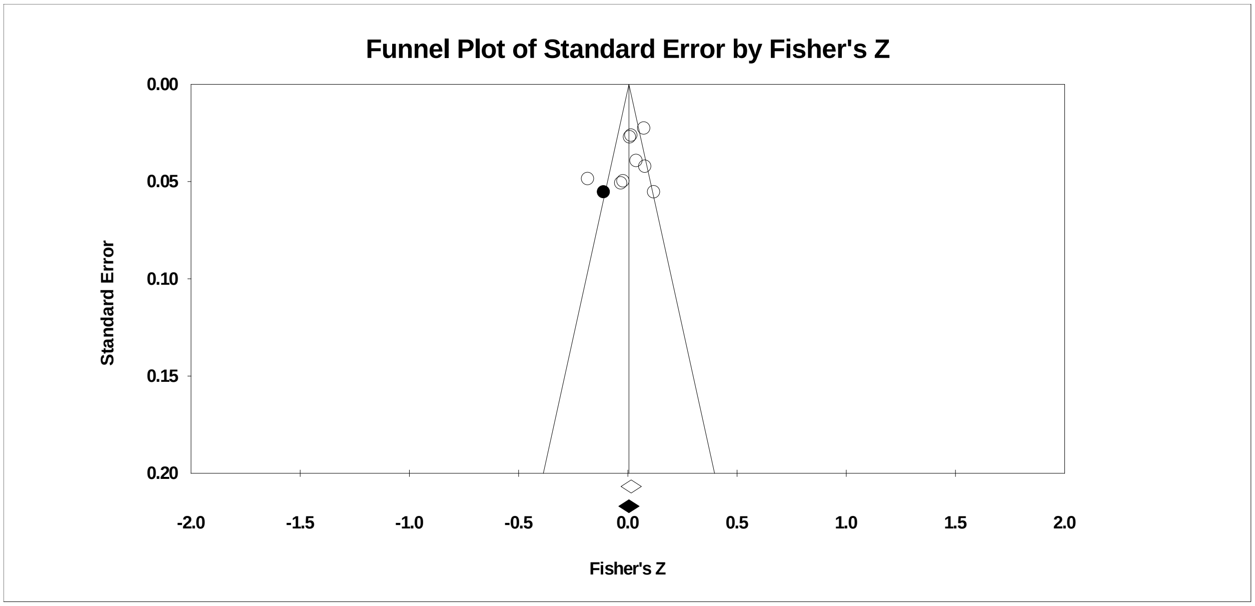

Figure S19. Task avoidance goal and negative emotions random-effects funnel plot.

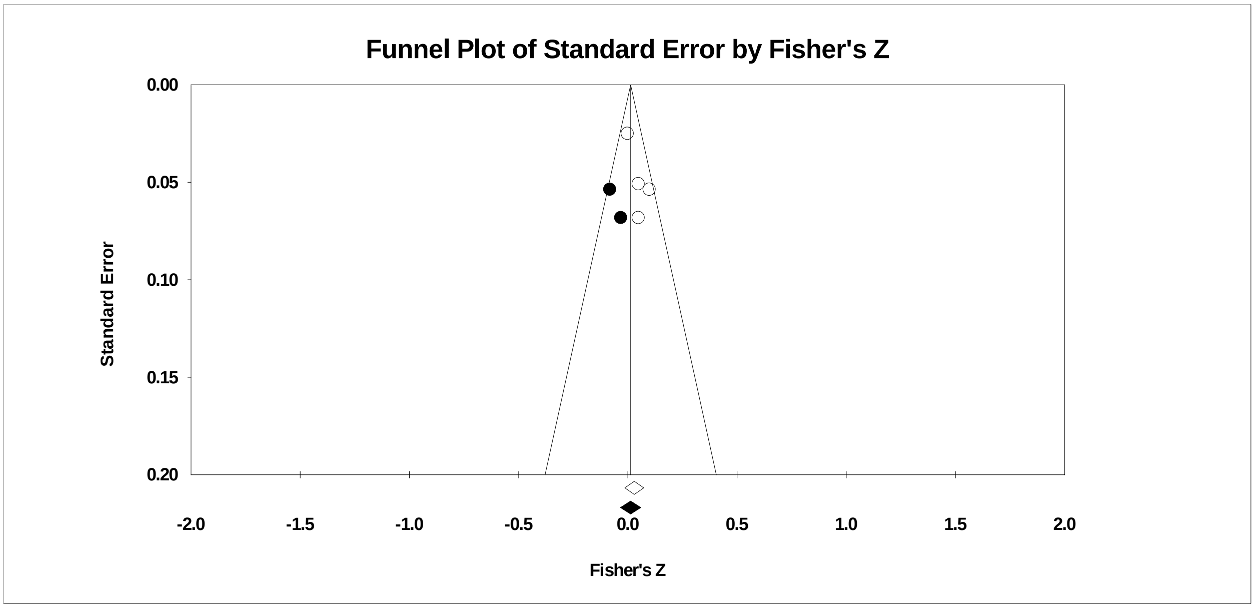

Figure S20. Task avoidance goal and performance random-effects funnel plot.

- Self- achievement goals and correlates.

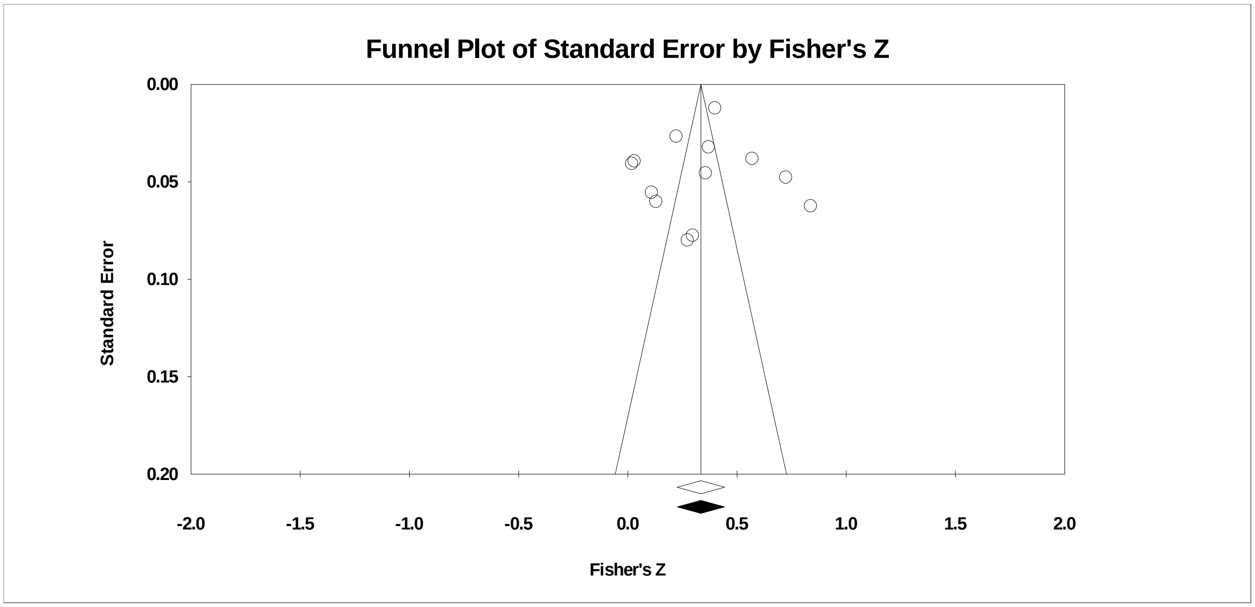

Figure S21. Self-approach goal and facilitative learning strategies random-effects funnel plot.

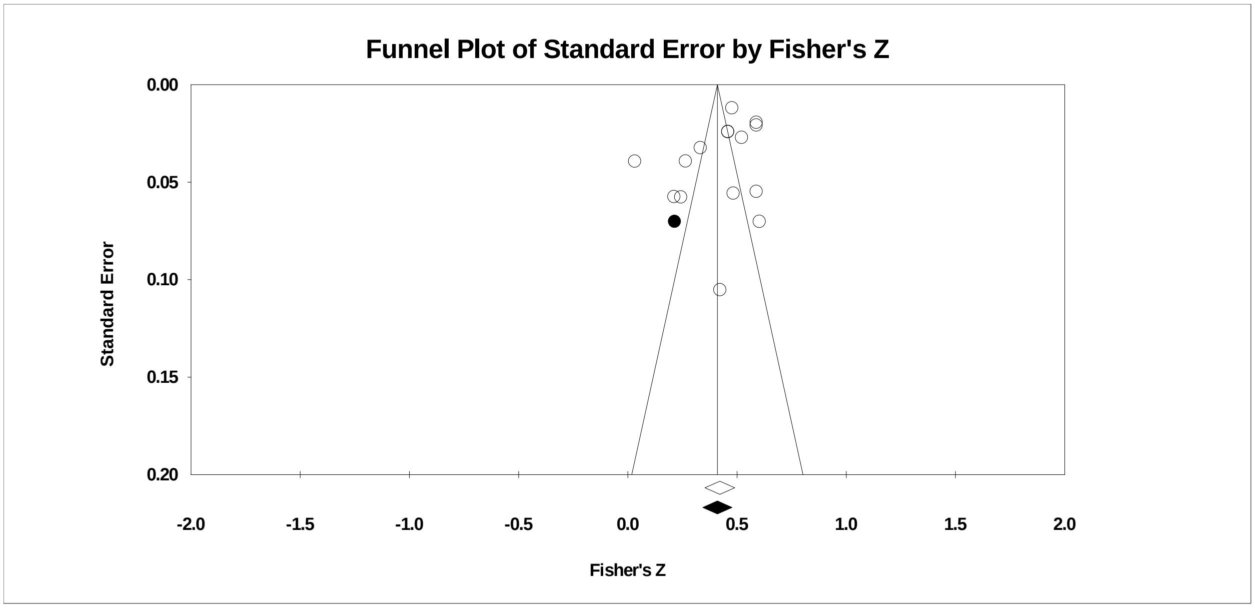

Figure S22. Self-approach goal and desired motivations random-effects funnel plot.

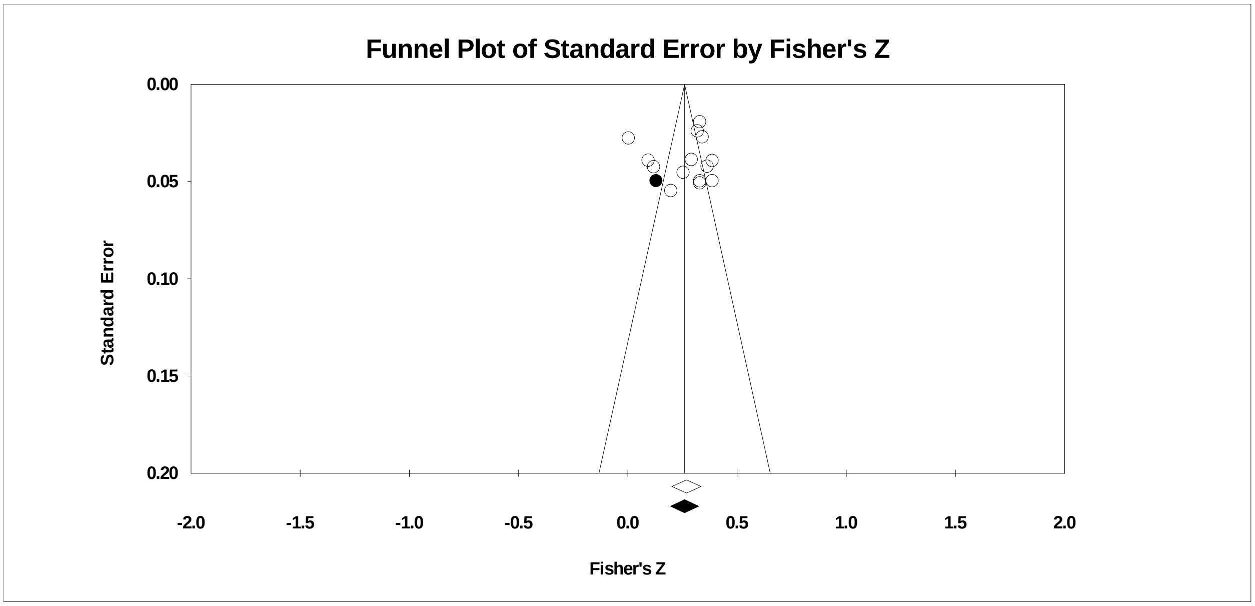

Figure S23. Self-approach goal and positive emotions strategies random-effects funnel plot.

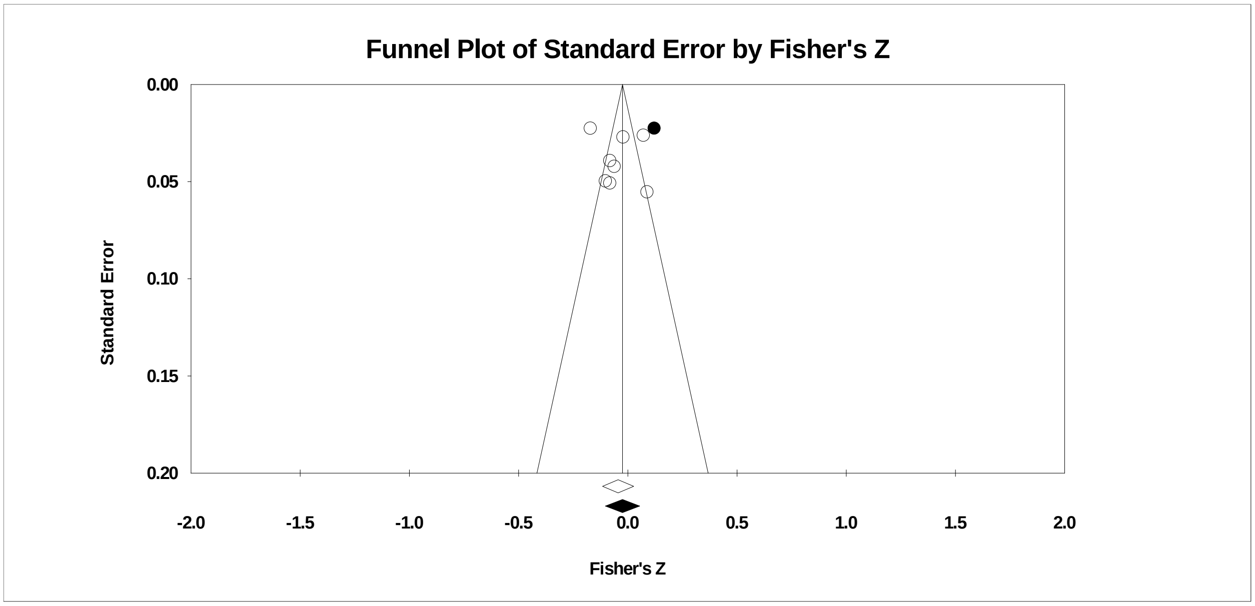

Figure S24. Self-approach goal and negative emotions random-effects funnel plot.

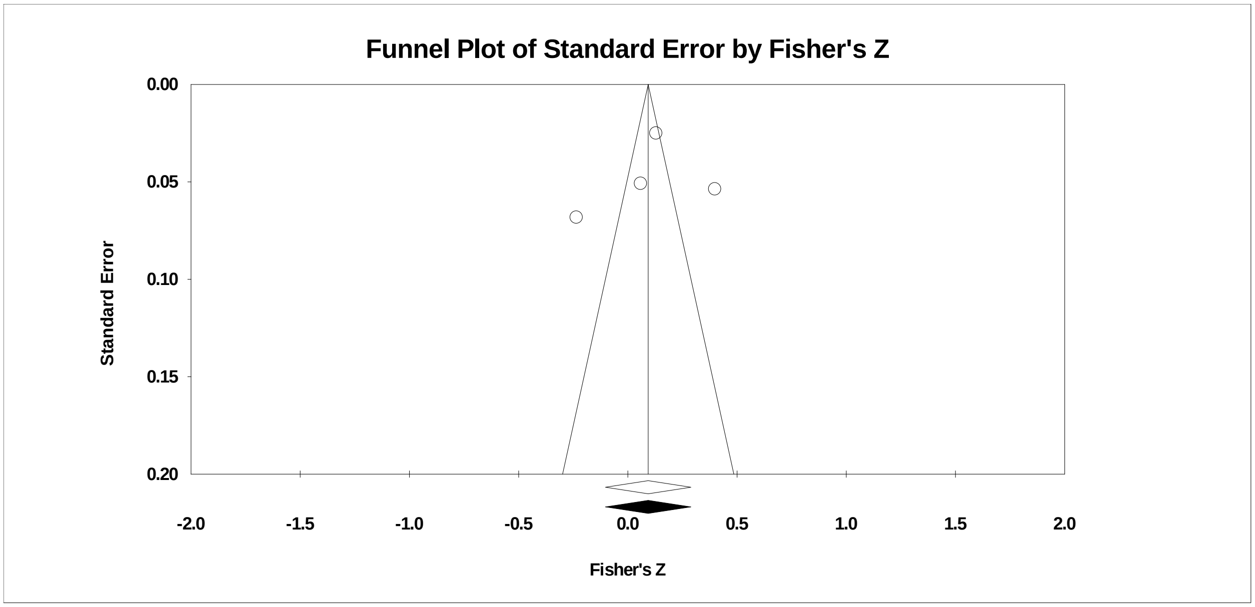

Figure S25. Self-approach goal and performance random-effects funnel plot.

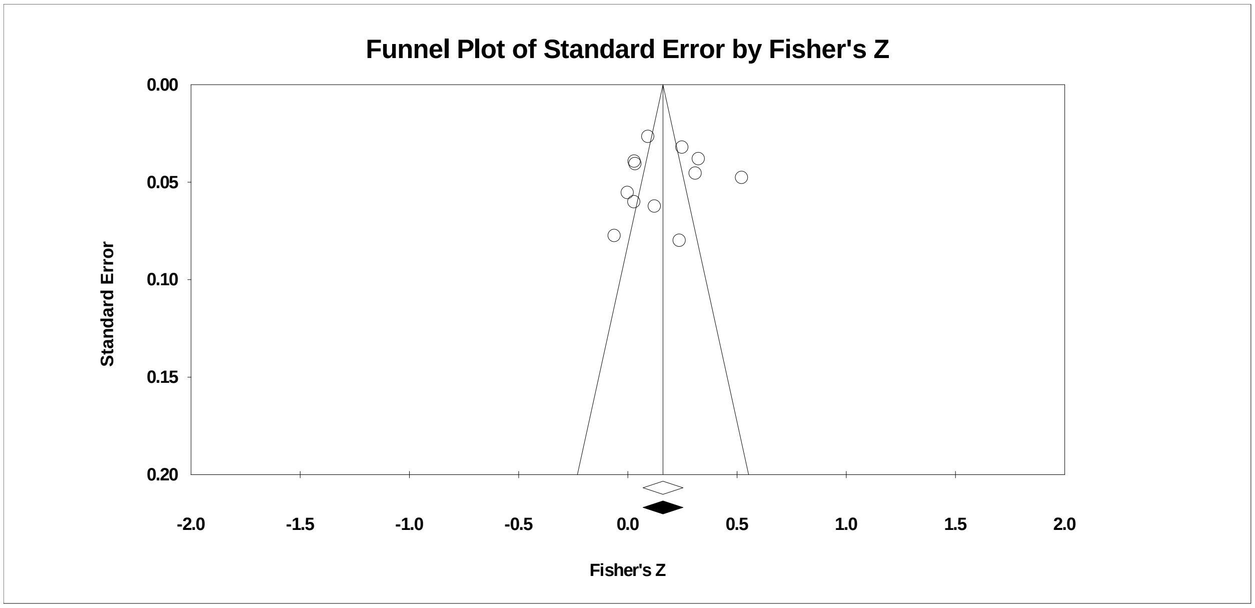

Figure S26. Self-avoidance goal and facilitative learning strategies random-effects funnel plot.

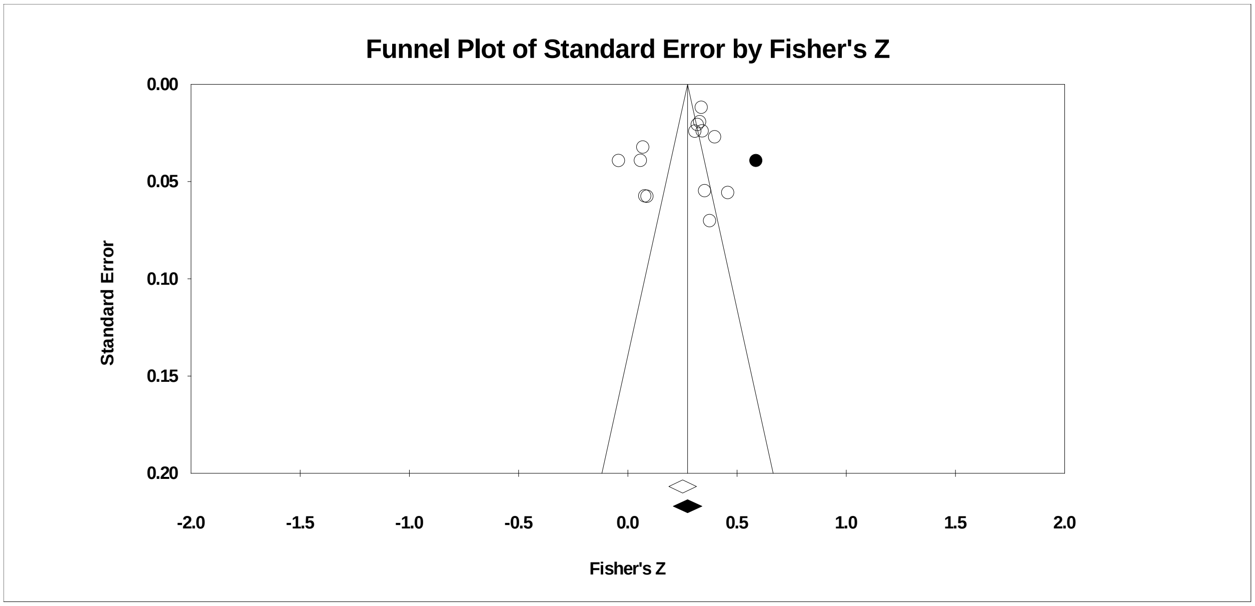

Figure S27. Self-avoidance goal and desired motivations random-effects funnel plot.

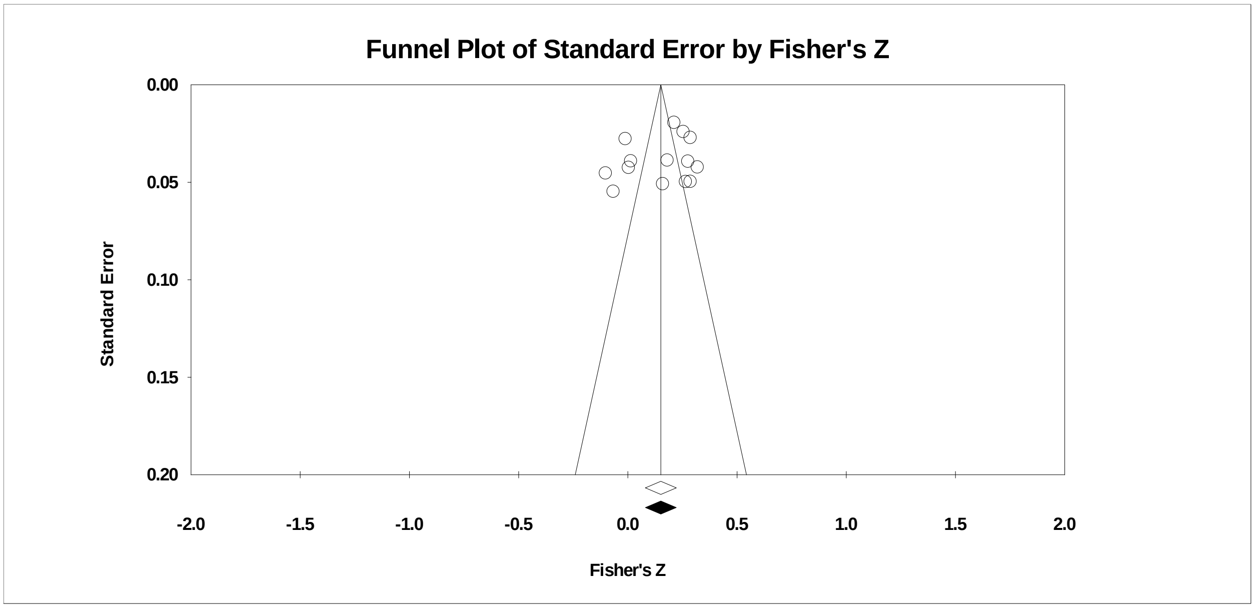

Figure Ss28. Self-avoidance goal and positive emotions strategies random-effects funnel plot.

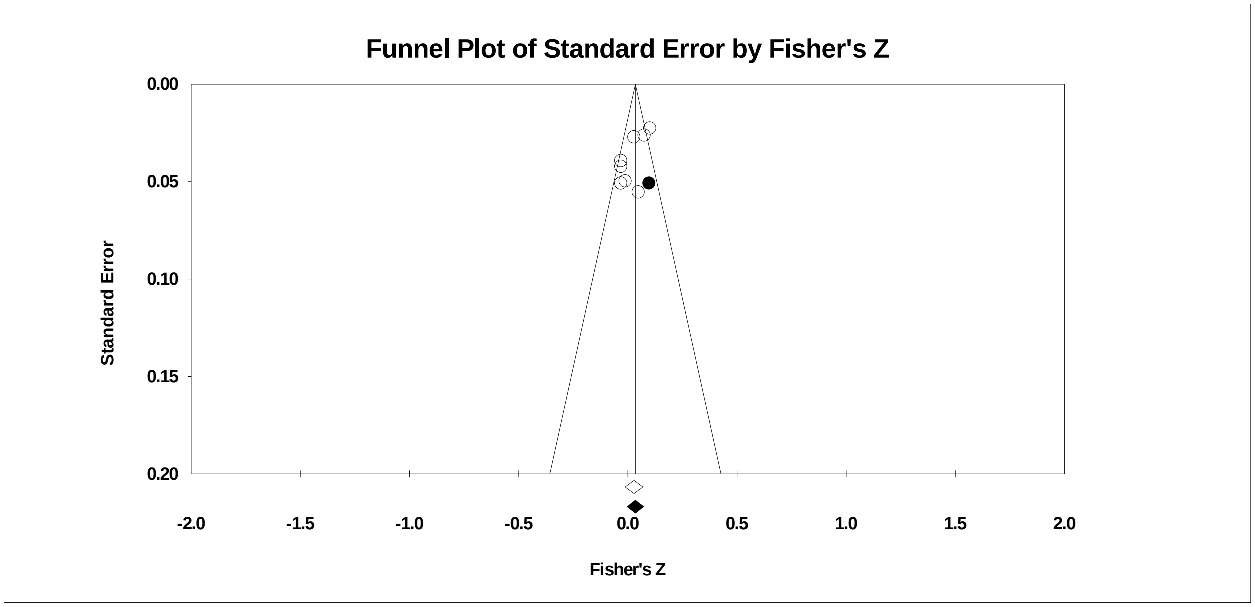

Figure S29. Self-avoidance goal and negative emotions random-effects funnel plot.

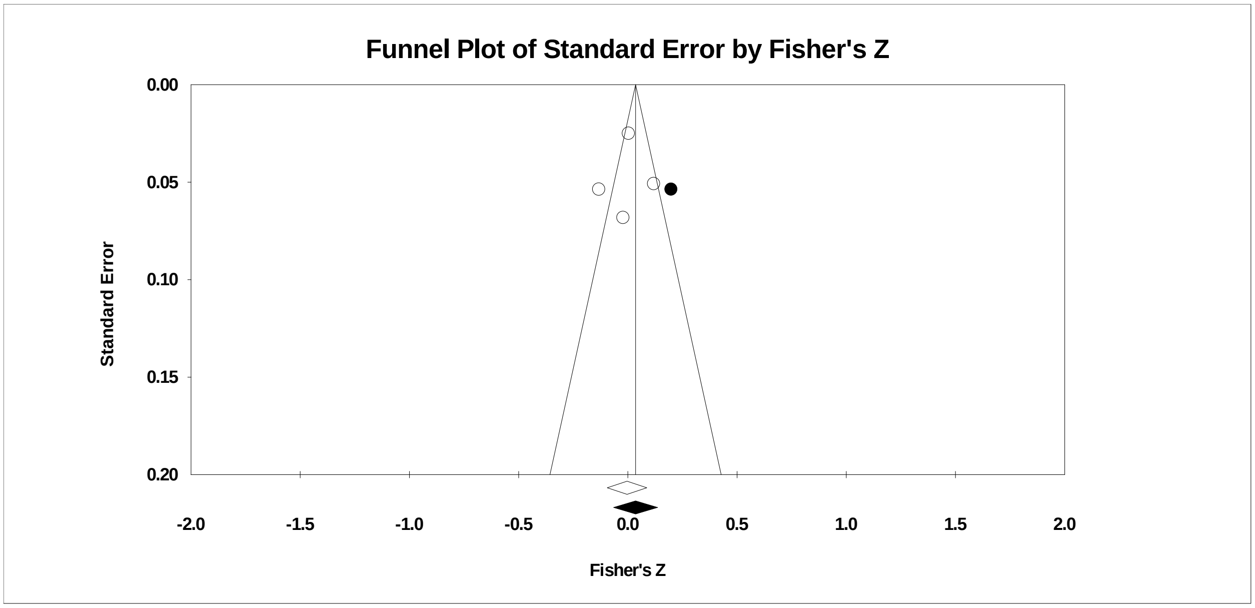

Figure S30. Self-avoidance goal and performance random-effects funnel plot.

- Other achievement goals and correlates.

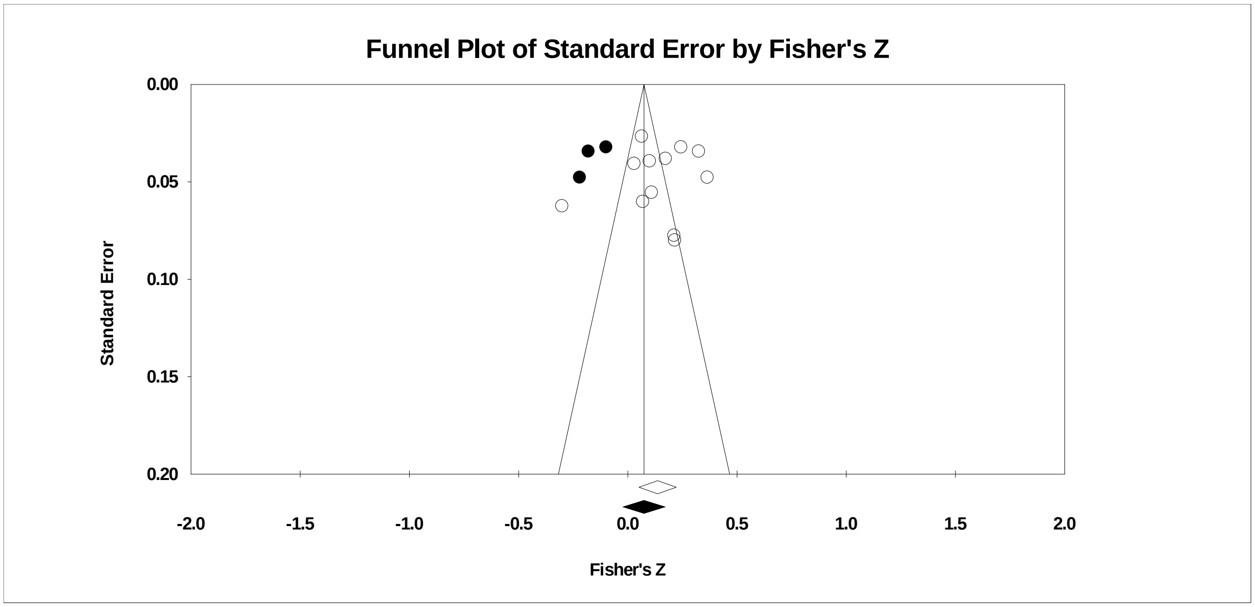

Figure S31. Other approach goal and facilitative learning strategies random-effects funnel plot.

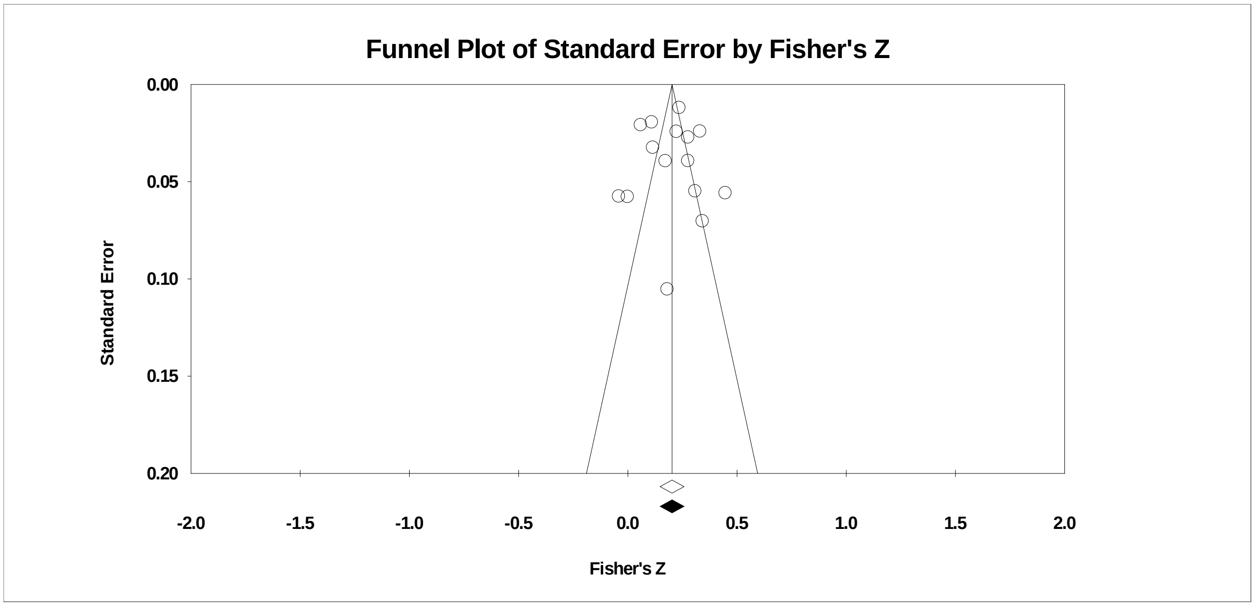

Figure S32. Other approach goal and desired motivations random-effects funnel plot.

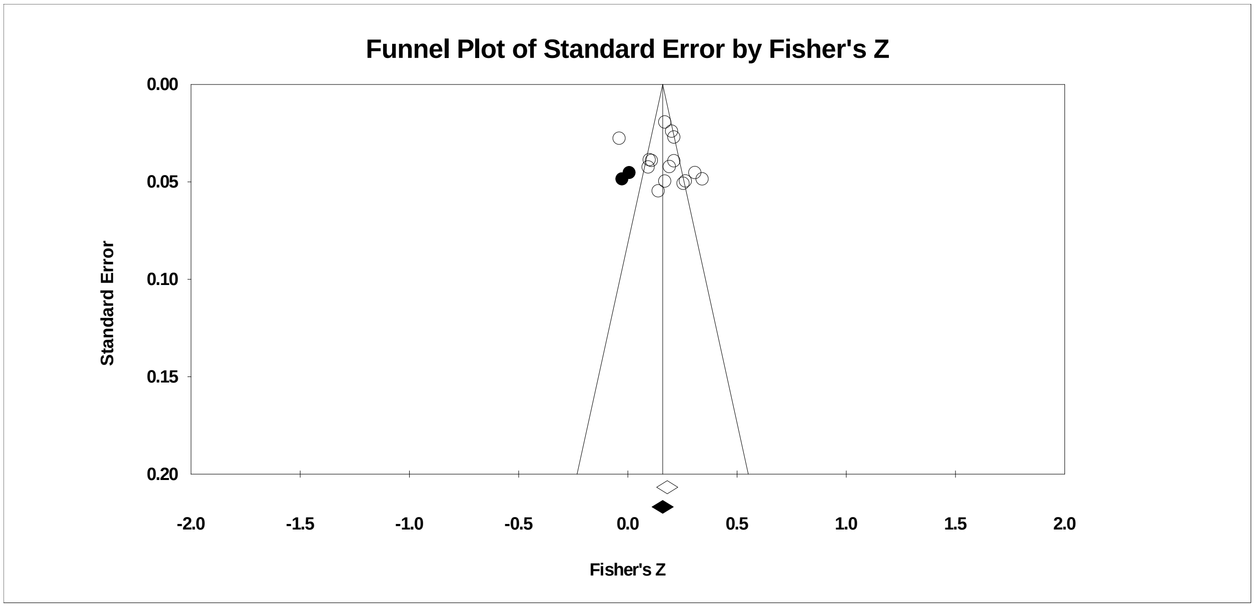

Figure S33. Other approach goal and positive emotions strategies random-effects funnel plot.

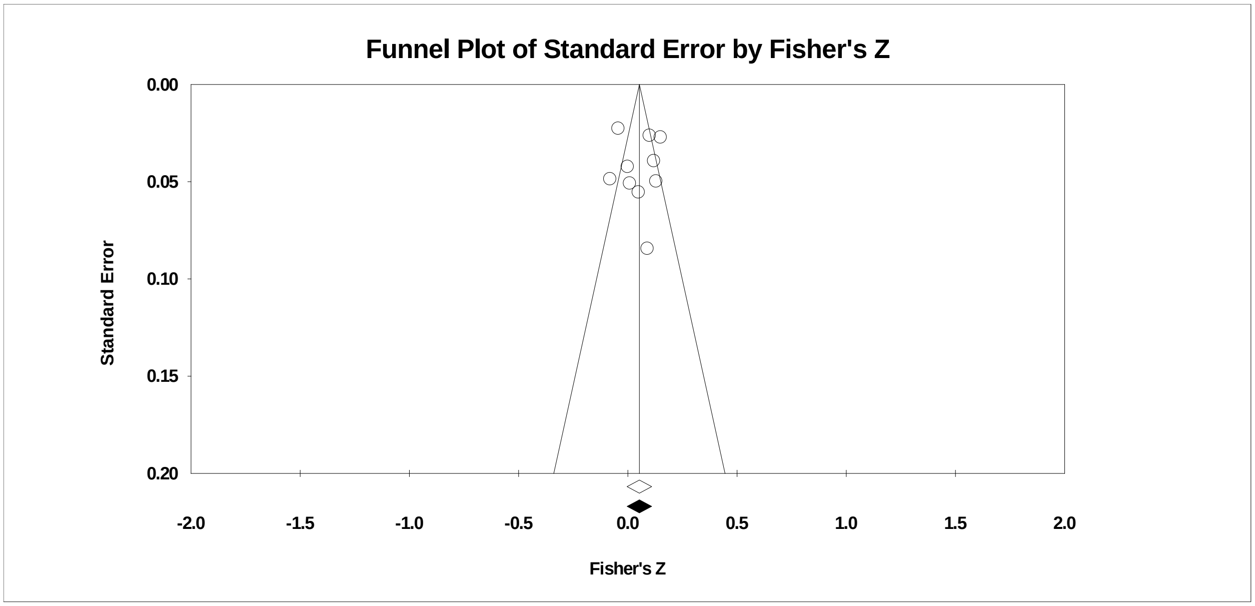

Figure S34. Other approach goal and negative emotions random-effects funnel plot.

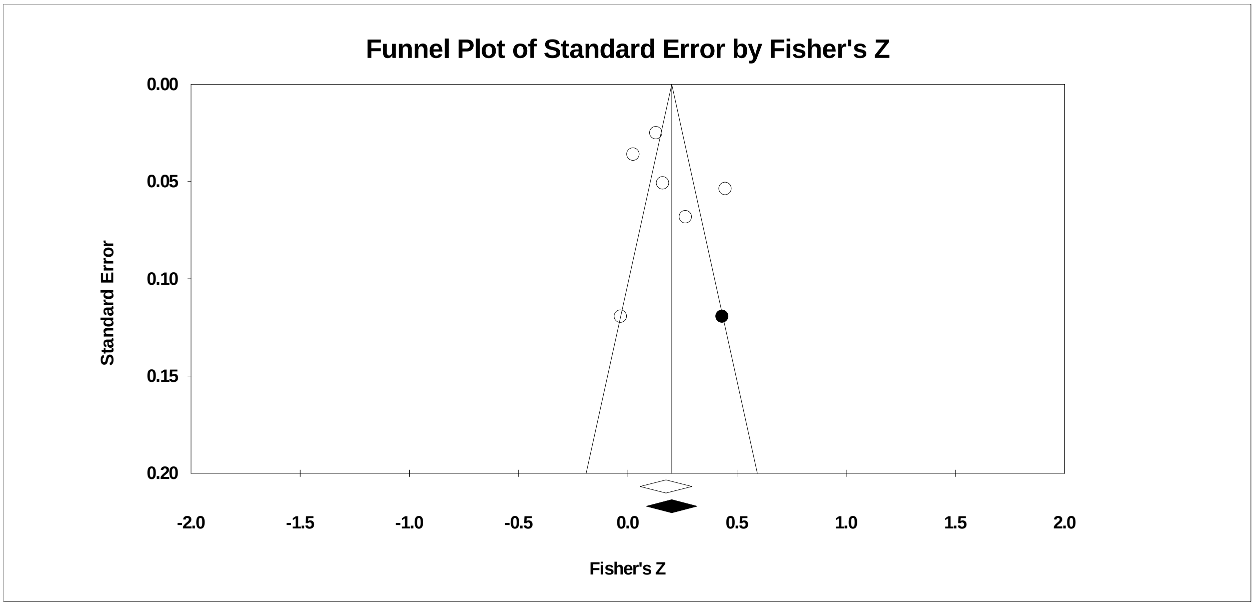

Figure S35. Other approach goal and performance random-effects funnel plot.

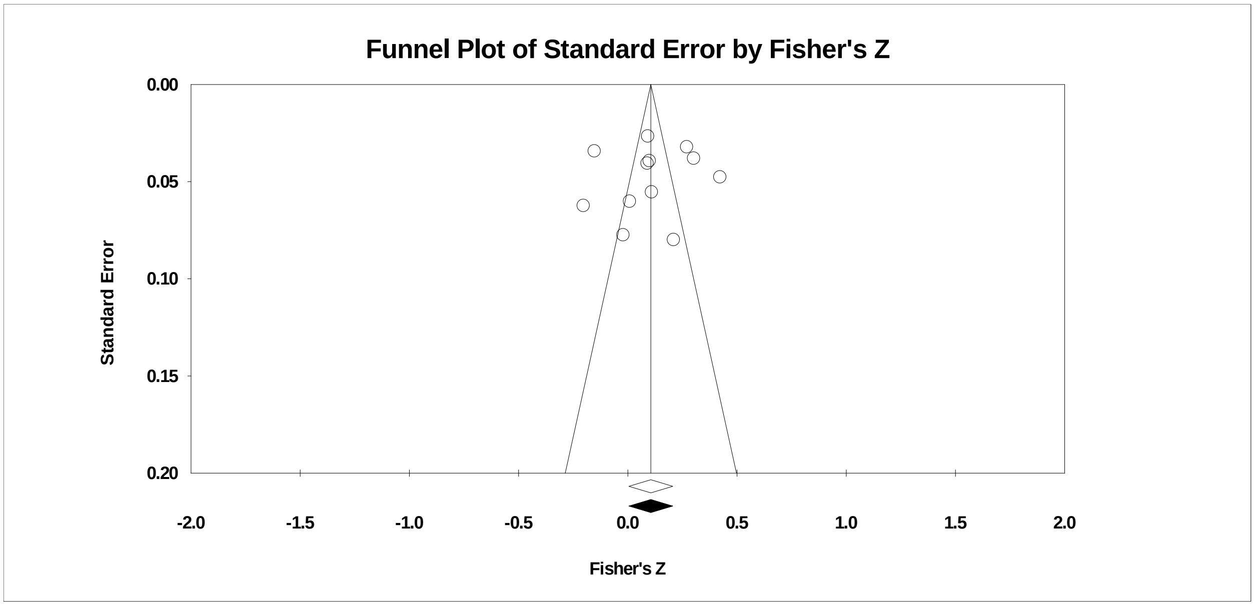

Figure S36. Other avoidance goal and facilitative learning strategies random-effects funnel plot.

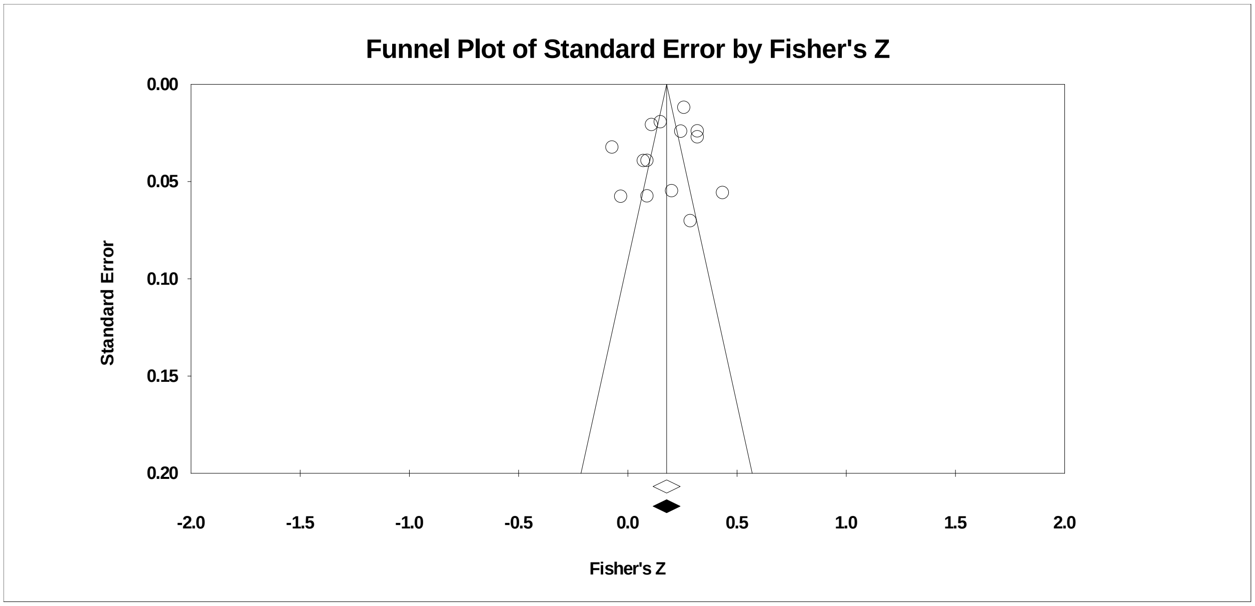

Figure S37. Other avoidance goal and desired motivations random-effects funnel plot.

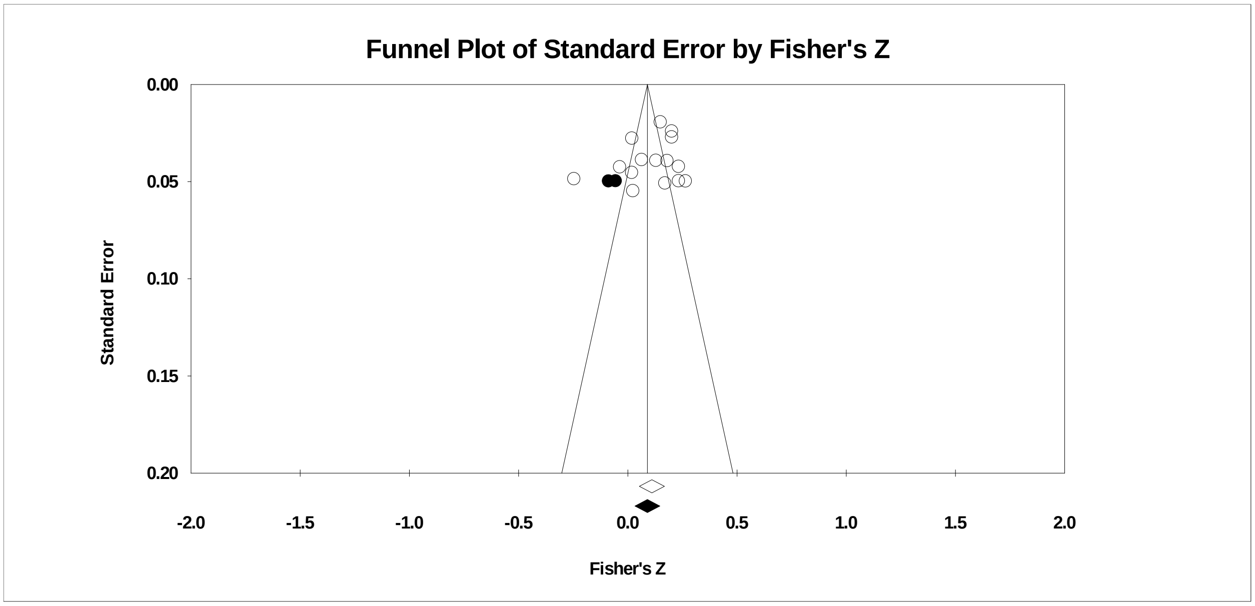

Figure S38. Other avoidance goal and positive emotions strategies random-effects funnel plot.

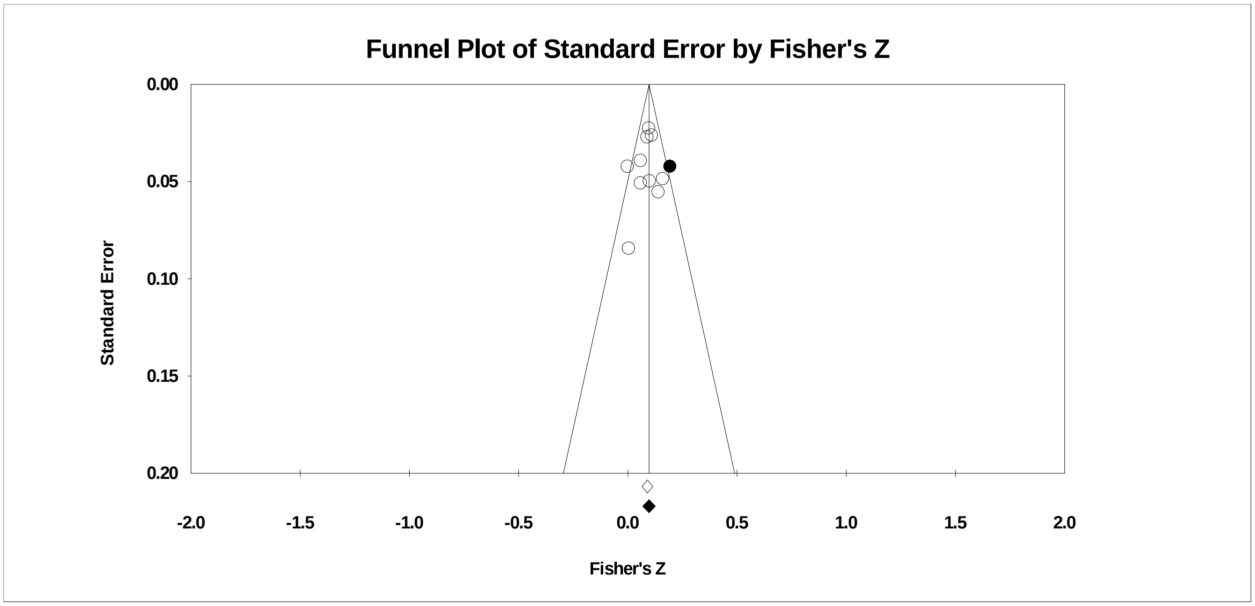

Figure S39. Other avoidance goal and negative emotions random-effects funnel plot.

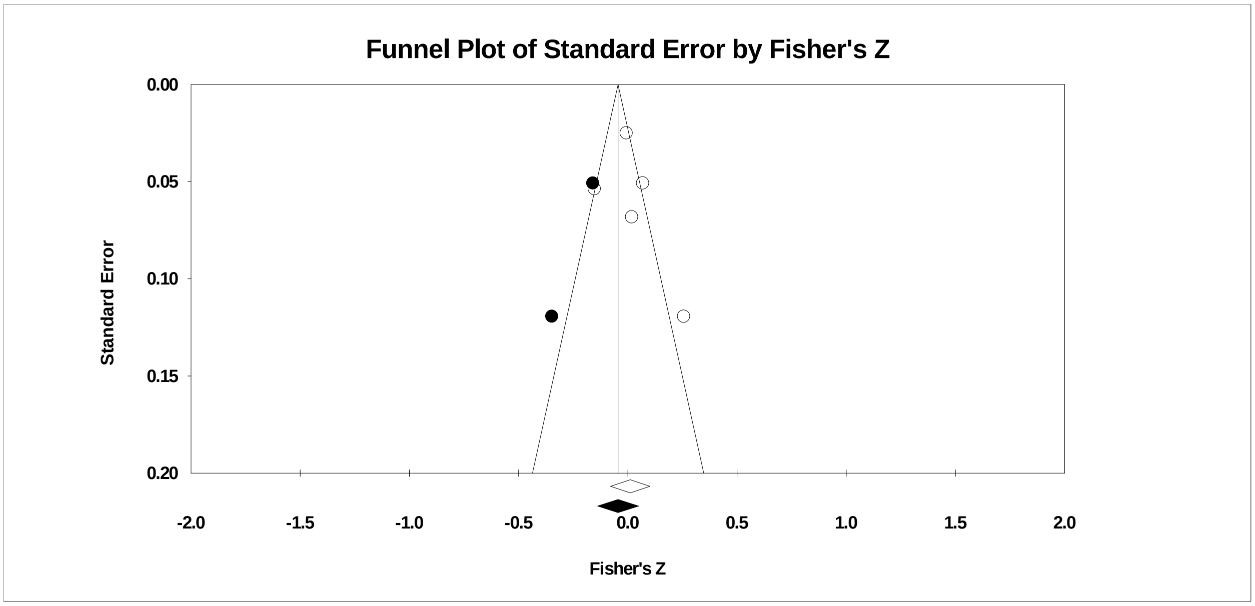

Figure S40. Other avoidance goal and performance random-effects funnel plot.
